# Elucidation of the key flavonol biosynthetic pathway in golden *Camellia* and its application in genetic modification of tomato fruit metabolism

**DOI:** 10.1093/hr/uhae308

**Published:** 2024-11-07

**Authors:** Lina Jiang, Leiqin Han, Wenxuan Zhang, Yifei Gao, Xiaoyan Xu, Jia Chen, Shan Feng, Zhengqi Fan, Jiyuan Li, Xinlei Li, Hengfu Yin, Pengxiang Fan

**Affiliations:** Department of Horticulture, Zhejiang University, Zijingang Campus, 866 Yuhangtang Road, Hangzhou 310058, China; Research Institute of Subtropical Forestry, Chinese Academy of Forestry, Hangzhou 311400, China; Department of Horticulture, Zhejiang University, Zijingang Campus, 866 Yuhangtang Road, Hangzhou 310058, China; Department of Horticulture, Zhejiang University, Zijingang Campus, 866 Yuhangtang Road, Hangzhou 310058, China; Department of Horticulture, Zhejiang University, Zijingang Campus, 866 Yuhangtang Road, Hangzhou 310058, China; Mass Spectrometry & Metabolomics Core Facility, The Biomedical Research Core Facility, Westlake University, Hangzhou 310030, China; Mass Spectrometry & Metabolomics Core Facility, The Biomedical Research Core Facility, Westlake University, Hangzhou 310030, China; Mass Spectrometry & Metabolomics Core Facility, The Biomedical Research Core Facility, Westlake University, Hangzhou 310030, China; Research Institute of Subtropical Forestry, Chinese Academy of Forestry, Hangzhou 311400, China; Research Institute of Subtropical Forestry, Chinese Academy of Forestry, Hangzhou 311400, China; Research Institute of Subtropical Forestry, Chinese Academy of Forestry, Hangzhou 311400, China; Research Institute of Subtropical Forestry, Chinese Academy of Forestry, Hangzhou 311400, China; Department of Horticulture, Zhejiang University, Zijingang Campus, 866 Yuhangtang Road, Hangzhou 310058, China; Key Laboratory of Horticultural Plants Growth and Development, Agricultural Ministry of China, Hangzhou 310058, China

## Abstract

Golden *Camellia* refers to a group of species in the genus *Camellia* that display yellow petals. The secondary metabolites in these petals hold ornamental significance and potential health benefits. However, the biosynthetic mechanisms governing the synthesis of these metabolites in golden petals remain elusive, and the exploitation of their bioactive components is not fully realized. This research involved the collection and analysis of 23 species of golden *Camellia*, leading to the discovery that flavonols, particularly quercetin 3-*O*-glucoside and quercetin 7-*O*-glucoside, are the primary contributors to the golden flower pigmentation. Integrative transcriptomics and coexpression network analyses pinpointed *CnFLS1* as a crucial gene in the biosynthetic pathway, which, in conjunction with *CnCHS*, *CnF3’H*, and *CnUFGT*, orchestrates the specific pathway for flower color development. The enzyme assays revealed a high affinity and catalytic efficiency of CnFLS1 for DHQ, and transient expression of *CnFLS1* in tobacco was shown to enhance the biosynthesis of quercetin flavonols, highlighting the pathway specificity in golden *Camellia*. Moreover, strategic transformations of cultivated tomatoes with various biosynthetic genes yielded transgenic lines exhibiting yellow fruit and quercetin-enriched flesh. These modified lines not only contained distinct flavonol components characteristic of golden *Camellia* but also demonstrated markedly improved antioxidant capabilities and enhanced resistance. The outcomes of this study not only elucidate the metabolic processes underlying the pigmentation of golden *Camellia* flowers but also provide a foundation for the development of novel tomato breeds through synthetic biology.

## Introduction

The genus *Camellia*, a member of the Theaceae family, comprises >260 species known for their striking floral displays and significant agricultural values. These plants are not only culturally and economically important but also renowned for their vibrant flower colors, which range widely across species, contributing to their horticultural allure and biodiversity. Among these *Camellia* species, most of them produce red flowers, while Sect. *Chrysantha* stands out due to its unique golden-yellow petals [1], a rare trait within the genus that has captured the interest of botanists and horticulturists alike. However, the rarity and beauty of the golden *Camellia* have also rendered them endangered, necessitating conservation efforts and scientific study of their distinctive pigmentation.

The pigmentation of golden *Camellia* flowers remains controversial among researchers. Early studies indicated that the petal pigments of golden *Camellia* were primarily flavonoids and carotenoids [2, 3]. Subsequent research has suggested that flavonoids are the predominant elements contributing to the yellow hue. Li et al. [4] analyzed the flavonoid composition of five species of golden *Camellia* flowers, pinpointing compounds such as quercetin 3-*O*-glucoside, quercetin 3-*O*-rutin, quercetin 7-*O*-glucoside, and kaempferol 3-*O*-glucoside as key pigment contributors. Conversely, other researchers, including Zhou et al [3] argue that carotenoids, such as violaxanthin, xanthophyll, and neoxanthin, play a pivotal role. These divergent views highlight the intricacy of plant pigment biosynthesis and the ongoing challenge to decode the molecular mechanism of floral coloration.

The biosynthetic pathway for these pigments remains less well studied in golden *Camellia* species. There were limited efforts to elucidate flavonoid biosynthesis in *Camellia nitidissima*, a typical species in Sect. *Chrysantha*, including the transcriptome sequencing analysis [3, 5] and cloning of several structural genes. Zhou [6] identified the chalcone synthase (CHS) and chalcone isomerase (CHI) from the *C. nitidissima* petals and found the expression of the two genes in tobacco resulted in minimal color alteration and a slight increase in flavonoid content [6, 7]. The flavonol synthase (FLS) gene of *C. nitidissima* overexpression can ultimately cause tobacco petals to turn white or light yellow, but it cannot produce a distinct yellow color, suggesting that other key biosynthetic enzymes are missing [8]. Further investigations have pointed to the involvement of genes such as dihydroflavonol reductase (DFR) and flavonoid 3′ -hydroxylase (F3’H) in flavonoid biosynthesis within *C. nitidissima* petals [9, 10]. Considering that multiple genes in the flavonoid pathway often act together to regulate flower coloration [11, 12], the key pathway responsible for the unique flower coloration in *C. nitidissima* has not been fully identified. The ongoing research requires more comprehensive understanding of the biosynthetic and regulatory landscape governing flavonoid biosynthesis and its role in golden *Camellia* floral color diversity.

Tomato is a staple vegetable in human diets and provides essential natural antioxidants, predominantly from the carotenoid lycopene [13, 14]. Flavonoids are another group of antioxidant nutrient found in many vegetables. In contrast to the lipophilic carotenoids, the hydrophilic nature of flavonoids renders them particularly suitable for fresh consumption and enhances their bioavailability within the human body [15]. However, in tomato fruits, the presence of flavonoids is mainly confined to the peel, while their concentration is exceptionally low and often barely detectable in the flesh [16, 17]. The challenge of increasing these compounds in tomato flesh has been a significant focus of genetic and molecular breeding programs. Notable efforts have been made to elevate the anthocyanin levels within tomato fruits, which has resulted in the creation of tomato varieties with purple or black hues [18, 19]. However, reports of significant increase in the flavonol content within the tomato flesh remain scarce.

In this study, we surveyed floral pigments from 23 species of golden *Camellia* and identified flavonols, predominantly quercetin 7-*O*-glucoside and 3-*O*-glucoside, as the key pigments responsible for the captivating golden-yellow coloration of *C. nitidissima* flowers. We then identified key genes involved in *C. nitidissima*’s flavonol biosynthesis and successfully reconstructed the pathway in both transient *Nicotiana benthamiana* and stably transformed tomato systems. These engineered tomatoes accumulated a diverse array of *C. nitidissima*-derived flavonols, including camelliaside A and camelliaside C. Our work not only provides insights into the unique yellow coloration of *C. nitidissima* but also serves as a promising model for horticultural crop and vegetable breeding, with the potential to enhance both nutritional value and consumer appeal.

## Results

### Flavonols, predominantly quercetin 7-*O*-glucoside and quercetin 3-*O*-glucoside, as key contributors to the yellow coloration of golden *Camellia* flowers

**Figure 1:**
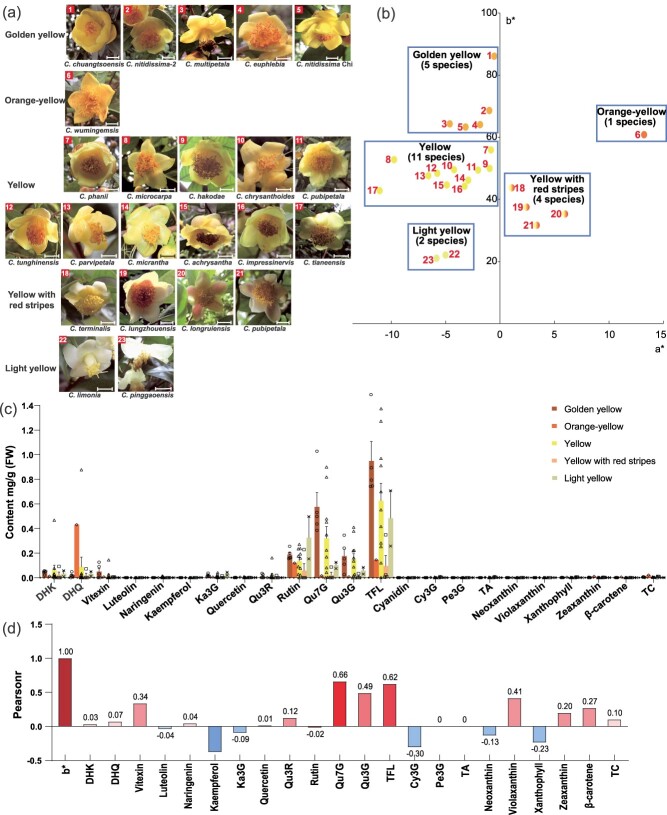
Identification of the predominant pigments in the petal coloration of golden *Camellia* through the analysis of 23 species. (a) Photographic representation of flowers from 23 species of golden *Camellia.* The scale bars in the figure represent a length of 1 cm. (b) Petal hue parameters plotted in a color space framework using chromaticity coordinates a* (The X axis) and b* (The Y axis). (c) Detailed quantification of flavonoid and carotenoid pigments in the petals of the 23 species of golden *Camellia*. Cy3G (cyanidin 3-*O*-glucoside), DHK (dihydrokaempferol), DHQ (dihydroquercetin), Ka3G (kaempferol-3-glucoside), Pe3G (pelargonidin-3-glucoside), Qu3G (quercetin 3-*O*-glucoside), Qu3R (quercetin 3-*O*-rutinoside), Qu7G (quercetin 7-*O*-glucoside), total flavonoids (TFL), total anthocyanins (TA), total carotenoids (TC). The presented data are mean ± SEM from three biological replicates. (d) Correlation analysis linking the color index hue b* (first column) with the measured pigment concentrations.

To investigate the primary pigments conferring the yellow hue in the petals of various *Camellia* species, we collected petals from 23 species of golden *Camellia* exhibiting a range of yellow color intensities (Fig. 1a). After quantitatively measuring the petal colors, we plotted the hue parameters of each sample within a color space framework, represented by the chromaticity coordinates a^*^ (red-green X axis) and b^*^ (yellow-blue Y axis), as illustrated in Fig. 1b. The hue a^*^ values spanned the red-green intersection, ranging from −10 to 5. Notably, the hue b^*^ values were exclusively situated in the yellow quadrant, with a variation from 20 to 90. Based on the observed color hues, we were able to categorize the petals of the 23 species of golden *Camellia* into five distinct chromatic groups: golden yellow, orange-yellow, yellow, yellow with red stripes, and light yellow.

To decipher the specific pigments responsible for the formation in yellow *Camellia* petals, two major groups of compounds, flavonoids and carotenoids, present within the petals were quantified via high-performance liquid chromatography (HPLC) analysis, the results were detailed in Supplementary Table S1. Intriguingly, our findings indicated a generally low concentration of carotenoids across the sampled yellow *Camellia* petals, except for the orange-yellow category, which contained a level of carotenoids. In contrast, there was a notable presence of flavonoid compounds, with significant variability in content. Flavonols, including quercetin 7-*O*-glucoside (Qu7G), quercetin 3-*O*-glucoside (Qu3G), and rutin, emerged as the predominant contributors to the yellow pigmentation, as evidenced in Fig. 1c. Another noteworthy observation is that the yellow with red stripes category contains a significant amount of cyanidin 3-*O*-glucoside (Cy3G), which is the primary pigment responsible for the red stripes in the petals. In non-yellow Camellia species with white, pink, red, and black petals, Cy3G emerged as the predominant pigment in the pink, red, and black varieties, whereas the white petals harbored only trace quantities of flavonols, such as quercetin glycosides (Supplementary Fig. S1).

Subsequently, we employed correlation analysis and stepwise multiple regression to distinguish the relationship between the hue b^*^ (yellowness index) and the concentrations of various flavonoid and carotenoid pigments. The correlation analysis, depicted in Supplementary Fig. S2, revealed a significant positive correlation of hue b^*^ with Qu7G (r = 0.659), Qu3G (r = 0.488), and the total flavonoids (TFL, r = 0.533), as shown in Fig. 1d. Moreover, the stepwise multiple linear regression model for hue b* can be represented by the following equation:

b^*^ = 43.217x1 + 13.411x2 + 74.648x3–22029.076x4–25209.869x5–551.379x6 + 36.459, with R^2^ = 0.955, where x1 corresponds to Qu7G, x2 to TFL, x3 to Qu3G, x4 to violaxanthin, x5 to kaempferol, and x6 to kaempferol 3-*O*-glucoside (Ka3G). Through iterative elimination of the less influential components, the refined stepwise regression model for the yellowness index (hue b^*^) retained three positive predictors, Qu7G, Qu3G, and TFL.

This analysis confirms that the yellowness index (hue b^*^) maintains a direct proportionality with the levels of Qu7G and Qu3G, thereby establishing them as the dominant factors influencing the yellow coloration of golden *Camellia* petals. The following research will focus on the identification of key genes responsible for the biosynthesis of the quercetin derivatives Qu7G and Qu3G.

### Dual-platform transcriptomic analysis unveils distinct petal-enriched genes and dynamic expression profiles in *C. nitidissima* flowers

**Figure 2:**
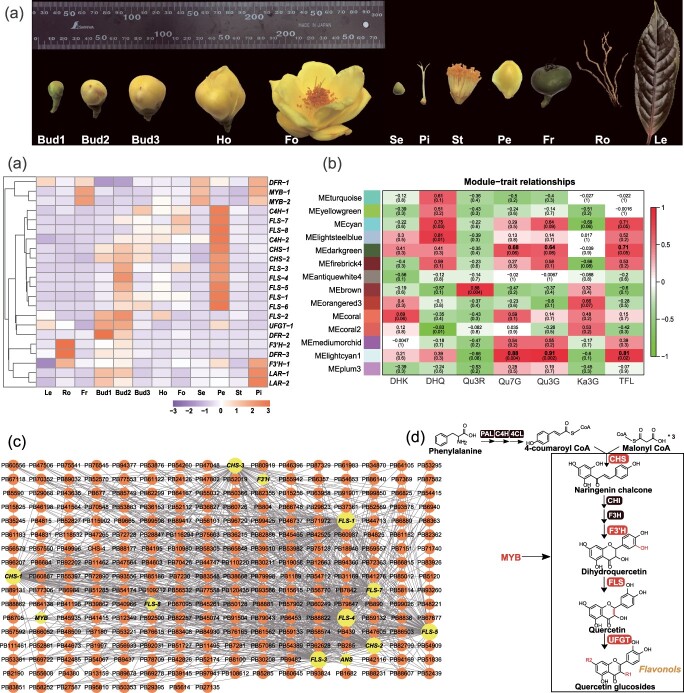
Dual-platform transcriptomic analysis identifies genes with significant correlations to the concentration of key yellow pigments in *C. nitidissima* petals. (a) Representative images of the 12 floral and non-floral *C. nitidissima* tissues subjected to transcriptomic sequencing. The samples, from left to right, include: Bud1 (10 mm diameter buds), Bud2 (20 mm diameter buds), Bud3 (30 mm diameter buds), Ho (half-opened flowers), Fo (fully opened flowers), Se (sepals), Pi (pistils), St (stamens), Pe (petals), Fr (fruits), Ro (roots), and Le (leaves). (b) Hierarchical clustering of flavonoid biosynthesis-related unigenes significantly correlated with the accumulation of Qu7G and Qu3G in *C. nitidissima* petals. (c) Module–trait relationships. The vertical axis represents 14 merged modules (with different colors). The horizontal axis represents the content of different pigments. Each cell within the grid shows the correlation coefficient and associated *P*-value between the module and pigment trait. (d) Visualization of the coexpression network linking gene modules to pigment traits, with weight values exceeding 0.25. Nodes in the network symbolize unigenes; key source genes pivotal to the network are labeled with gene names in the diagram. (e) Schematic diagram of the proposed metabolic pathway responsible for the formation of the golden yellow coloration in *C. nitidissima* petals. This diagram integrates the findings from the WGCNA, suggesting a biosynthetic pathway catalyzed by several key enzymes (highlighted with increase font size) is involved in the biosynthesis of the characteristic yellow flower pigment flavonols.

In order to identify the genes responsible for the biosynthesis of flavonols that render the yellow hue to golden *Camellia* petals, we selected *C. nitidissima* for in-depth transcriptomic analysis. We collected *C. nitidissima* flowers at five developmental stages: 10-mm buds, 20-mm buds, 30-mm buds, half-opened flowers, and fully opened flowers; various floral organs: sepals, stamens, and pistils; and several non-floral tissues: roots, leaves, and fruits to perform a comprehensive transcriptomic analysis (Fig. 2a).

Due to the absence of *C. nitidissima* reference genome, we utilized dual-sequencing platforms to comprehensively capture its full-length transcripts and identify differentially expressed genes (DEGs). Following the generation of full-length transcripts through PacBio sequencing, Illumina RNA-seq was conducted on selected *C. nitidissima* tissues for DEG analysis across 12 tissues. This investigation uncovered 67 018 DEGs, with 25 986 being annotated within the Kyoto Encyclopedia of Genes and Genomes (KEGG) database. The most substantial number of DEGs was recorded in the 20-mm buds (Bud 2) and half-opened flowers, totaling 27 186 (Supplementary Fig. S3b). DEGs increased from the bud stage to the half-open phase, reaching a peak of 21 012 DEGs in half-opened flowers, before declining to the lowest number of 5 063 in the fully opened flowers (Supplementary Fig. S3c). This pattern highlights the most substantial transcriptomic shifts occurring as the bud progresses to the half-opened stage. Additionally, a comparative analysis of various floral tissues revealed a market difference in DEG profiles between the petals and other floral parts, including stamens, pistils, and sepals (Supplementary Fig. S3d). This suggests that the petals have unique gene expression patterns that likely contribute to their coloration and other specialized functions.

### Multiomics approach unveils coexpression networks and candidate genes controlling flavonol biosynthesis in *C. nitidissima* petal*s*

We further combined HPLC with transcriptomic analysis to identify genes governing the biosynthesis of yellow flavonol pigments in *C. nitidissima* petals. Firstly, a correlative analysis was conducted between the contents of Qu7G and Qu3G, the key contributors to the yellow hue of *Camellia* flower, and DEGs based on their fragments per kilobase of transcript per million mapped reads (FPKM) values in the 12 different *C. nitidissima* tissues. We identified an array of unigenes significantly correlated with Qu7G, Qu3G (*P* < .0.05) and annotated them in the flavonoid biosynthetic pathway (detailed in Supplementary Table S2). These included genes critical for early steps (CHS; Cinnamate 4-hydroxylase, C4H), genes shaping the flavonol (FLS, F3’H) and anthocyanin (DFR; leucoanthocyanidin reductase, LAR) backbones, also flavonoid glycosylation enzyme (UFGT) and MYB transcription factors regulating the pathway (Supplementary Fig. S4). Developmentally, these unigenes peaked in Bud 1 or Bud 2 stage and gradually declined as flowers opened, mirroring the dynamic accumulation of yellow pigments (Fig. 2b). We therefore propose C4H, FLS, CHS, F3’H, and UFGT as candidate genes for yellow flower pigments in *C. nitidissima.*

Weighted gene coexpression network analysis (WGCNA) was employed to further refine candidate genes associated with yellow pigment formation. After filtering low-expression genes, 22 658 KEGG-annotated DEGs (out of 25 986) were subjected to analysis (detailed in Supplementary Data S1). Sample clustering (Supplementary Fig. S5a) based on gene expression levels can effectively correlate genes with traits (the contents of pigment detailed in Supplementary Data S2) and selection of the soft-thresholding power was critical, power = 10 was found to construct the network (Supplementary Fig. S5b, c). Utilizing the dynamic tree-cutting method, we identified 90 modules and subsequently merged similar modules, culminating in a total of 14 distinct coexpression modules (Supplementary Fig. S5d). The Turquoise module was the most gene-rich (containing 4996 genes); the module Lightsteelblue was the smallest, containing 107 genes (Supplementary Fig. S5e). We investigated the correlations between the obtained gene modules and the respective flavonoid and carotenoid pigments. The analysis revealed a strong correlation between the principal yellow flower pigments (Qu7G and Qu3G) and module Lightcyan1 (r = 0.88, P = 0.004 for Qu7G; r = 0.91, P = 0.002 for Qu3G). Additionally, module Darkgreen displayed a notable association with the primary yellow pigments (r = 0.68 for Qu7G; r = 0.64 for Qu3G), although the correlations were not statistically significant (Fig. 2c). Within modules Lightcyan1 and Darkgreen, we identified 12 unigenes involved in the flavonoid biosynthesis (detailed in Supplementary Data S3). The gene coexpression network highlighted *FLS* as the central hub gene, with significant connections to CHS, F3’H, and the MYB transcription factor suggesting these as coexpressed genes (Fig. 2d). After performing BLAST searches against the NCBI databases, these genes were identified as follows: *CnFLS1*, JF1343560.1; *CnCHS*, HQ269804.1; *CnF3’H*, HQ290518.1; and *CnMYB111*, MT370521.1.

Collectively, the above correlation analysis revealed four flavonoid biosynthetic genes and an *MYB* transcription factor as predominant component for the biosynthesis of the primary flavonol pigments contributing to the yellow color of *C. nitidissima* petals (Fig. 2e).

### The FLS from *C. nitidissima* demonstrates a superior catalytic efficiency in converting dihydroquercetin to quercetin compared to FLSs from other plants

We established that quercetin-derived flavonols, Qu7G and Qu3G, are the principal pigments conferring the golden yellow hue to *Camellia* flowers. The FLS enzyme plays a crucial role in flavonol biosynthesis, transforming flavanonols such as dihydrokaempferol (DHK) and dihydroquercetin (DHQ) into flavonols by introducing a double bond into the C-ring of the flavonoid structure. FLS emerged as a pivotal gene in our WGCNA, significant in driving the biosynthesis of flavonols in golden *Camellia* petals. Thus, we postulate that CnFLS1 operates in the petals of *C. nitidissima* to catalyze the conversion of flavanonols like DHQ into quercetin, subsequently transformed into Qu7G and Qu3G.

**Figure 3:**
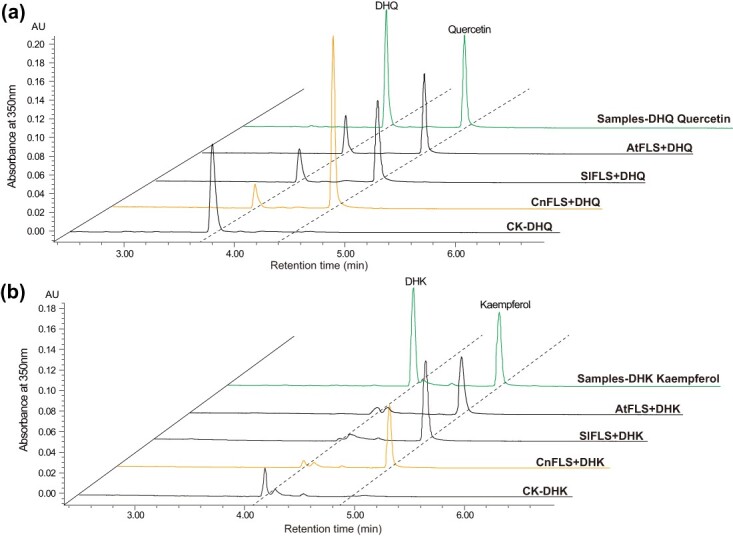
Assessment of the catalytic efficiency of recombinant FLS enzymes in the biosynthesis of flavonols. (a) HPLC analysis of the products from the enzymatic reaction of recombinant FLS proteins using DHQ as a substrate. DHQ serves as the precursor molecule that is converted into quercetin by the FLS enzyme. The chromatogram labeled with “CnFLS+DHQ” represents the reaction mixture containing CnFLS1 enzyme and DHQ, while the chromatogram labeled with “Sample-DHQ Quercetin” depicts the reference standard of DHQ and quercetin. The analysis shows that all three FLS enzymes can convert DHQ to quercetin, with CnFLS1 demonstrating the highest catalytic activity toward this substrate. (b) HPLC analysis of the enzymatic reaction products of recombinant FLS proteins with DHK as the substrate. DHK is transformed into kaempferol through the action of FLS. The chromatographic labeled with “CnFLS+DHK” represents the reaction mixture with CnFLS1 enzyme and DHK, and the chromatogram labeled with “Sample-DHK Kaempferol” represents the DHK and kaempferol standard. The results indicate that all three recombinant enzymes are capable of converting DHK to kaempferol.

To investigate whether the CnFLS1 enzyme in *C. nitidissima* exhibits high catalytic performance toward DHQ, we measured the enzyme kinetics of CnFLS1 using DHK and DHQ as substrates and compared it with the kinetics of FLS1 proteins from tomato (SlFLS) and Arabidopsis (AtFLS1) (Supplementary Fig. S6). The assessments revealed all three enzymes were active on both DHQ and DHK substrates (Fig. 3). Notably, CnFLS1 displayed a stronger affinity for DHQ (*Km* = 18.68 μM) over DHK (*Km* = 60.63 μM) and exhibited a lower *Km* than both SlFLS (26.51 μM) and AtFLS1 (29.89 μM), as shown in Supplementary Fig. S6 and Table 1. This suggests CnFLS1 has the highest substrate affinity for DHQ among the enzymes tested. Moreover, CnFLS1’s catalytic efficiency for DHQ was also the most notable, with a *kcat/Km* of 1318.7 M^−1^ s^−1^, leading to a significantly enhanced biosynthesis rate of quercetin compared to SlFLS (702.88 M^−1^ s^−1^) and AtFLS1 (794.02 M^−1^ s^−1^) (Fig. 3a, Table 1).

**Table 1:** Kinetic parameters of recombinant FLS proteins from multiple plant species.

Species	Enzyme	DHQ			DHK		
		*Km* (μM)	*kcat* (s-1)	*kcat/Km* (M-1 s-1)	*Km* (μM)	*kcat* (s-1)	*kcat/Km* (M-1 s-1)
*C. nitidissima*	CnFLS1	18.68	0.02	1318.70	60.63	0.08	1302.16
*S. lycopersicum*	SlFLS	26.51	0.02	702.88	40.45	0.08	1899.88
*A. thaliana*	AtFLS1	29.89	0.02	794.02	95.01	0.11	1179.35
*C. sinensis* [20]	CsFLSc	34.56	0.02	617.05	27.11	0.02	637.82
*A. cepa* [21]	AcFLS	24.43	0.01	392.36	20.32	0.00	69.28
*Z. mays* [22]	ZmFLS1	151.10	3.90	27.40	58.40	6.60	111.80

An extensive literature survey revealed that the known plant FLS enzymes possess less affinity and catalytic efficiency for DHQ compared to CnFLS1. We found that the affinity and catalytic efficiency of various known plant FLS enzymes for DHQ are lower than those of CnFLS1. For example, the tea tree (*Camellia sinensis*) enzyme CsFLSc [20] has a *Km* for DHQ of 34.56 μM, nearly twice that of CnFLS1, while its *kcat/Km* is only 617.05 M^−1^ s^−1^, half that of CnFLS1. The FLS enzymes from *Allium cepa* and *Zea mays* exhibit even lower catalytic efficiency for DHQ, with *kcat/Km* of AcFLS at 392.35 M^−1^ s^−1^ [21] and *kcat/Km* of ZmFLS1 at 27.40 M^−1^ s^−1^ [22], respectively (Table 1).

These results suggest that CnFLS1 efficiently uses DHQ as a substrate and converts it into quercetin, which can be further glycosylated into the major pigment of golden *Camellia* flowers. The next step is to investigate how other key genes, such as CnCHS, CnF3’H, and CnMYB111, work together with CnFLS1 to govern flower pigmentation in *C. nitidissima*.

### Heterologous transient expression and recapitulation of flavonol biosynthetic pathway in *N. benthamiana*

To illuminate the functions of the identified genes, we initially generated phylogenetic trees comprising these *C. nitidissima* genes alongside homologous sequences from a diverse range of plant taxa (Supplementary Fig. S7). The analysis revealed that these *C. nitidissima* genes cluster closely with established flavonoid structural genes from a wide range of plant families. This insight led us to propose that the flavonol pigments of *C. nitidissima* could be recapitulated in *N. benthamiana* through the heterologous transient expression. Furthermore, due to concerns that cis-regulatory elements may have diverged significantly between Theaceae and Solanaceae, we opted to assess the tomato homolog *SlMYB12* (Solyc01g079620) as a proxy. The close evolutionary relationship between *CnMYB111* and *SlMYB12* (Solyc01g079620) (Supplementary Fig. S8) allows us to infer the function of *CnMYB111* by examining the effects of *SlMYB12* expression.

**Figure 4:**
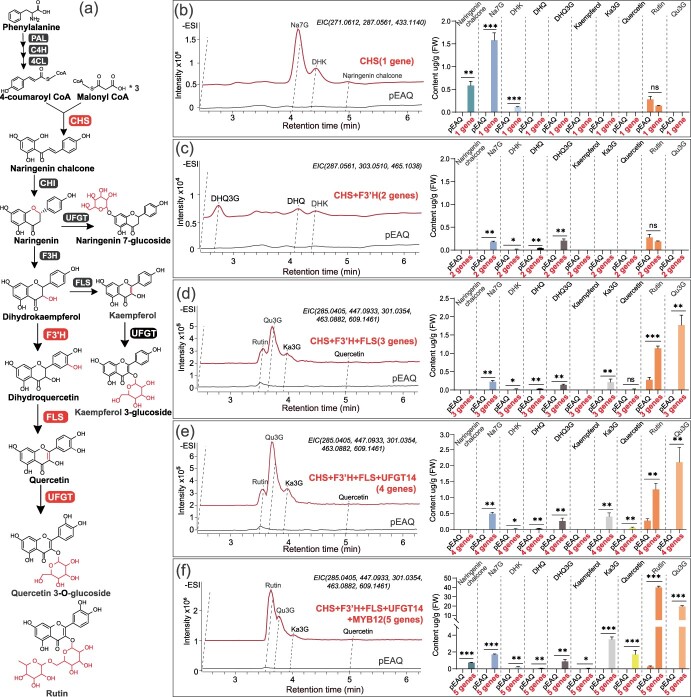
Reconstruction of *C. nitidissima* flavonol biosynthetic pathway in *N. benthamiana* leaves. (a) Schematic diagram detailing the biosynthetic pathway for quercetin production in *C. nitidissima*. The enzymes that are critical for this pathway and were used for the reconstruction in *N. benthamiana* are highlighted with increased font size. (b) to (f) The Extracted Ion Chromatograms (EIC) for the primary flavonoid molecules synthesized in *N. benthamiana* leaves post-transient expression of various combinations of candidate genes from *C. nitidissima*. The EIC provides a graphical representation of the abundance of a specific ion (which corresponds to a particular compound) across the chromatographic run time, which aids in quantifying the compounds. Adjacent to each panel is the quantification of the respective flavonoid compounds. The gene combinations used to achieve these metabolic constructs are shown as follows using empty vector control (labeled pEAQ) as the control: (b) CnCHS gene alone (labeled CHS); and (c) together with CnF3’H gene (labeled CHS+F3’H). (d) CnCHS, CnF3’H, and CnFLS1 genes combined (labeled CHS+F3’H+FLS). (e) CnCHS, CnF3’H, CnFLS1, and CnUFGT14 genes combined (labeled CHS+F3’H+FLS+UFGT14). (f) The full pathway reconstruction including CnCHS, CnF3’H, CnFLS1, CnUFGT14, and SlMYB12 transcription factor (labeled CHS+F3’H+FLS+UFGT14+MYB12). The compounds are represented by their respective mass-to-charge ratio (m/z) values within parentheses. The flavonoids profiled include Na7G (naringenin 7-*O*-glucoside), DHK (dihydrokaempferol), DHQ (dihydroquercetin), DHQ3G (dihydroquercetin 3-glucoside), Ka3G (kaempferol-3-glucoside), and Qu3G (quercetin 3-*O*-glucoside). The data shown are mean ± SEM from three biological replicates, with each plant infiltration considered a separate biological sample. Statistical significance in the levels of flavonoid compounds between the control (pEAQ vector) and the gene expression treatments was calculated using an unpaired *t*-test, with asterisks indicating the significance level (^*^P < 0.05, ^**^P < 0.01, ^***^P < 0.001).

To capture intermediate metabolites (Supplementary Fig. S9, S10) produced by each gene and dissect their individual contributions to *C. nitidissima* flavonol biosynthesis, we sequentially combined these genes based on their predicted catalytic order. Five distinct combinations were prepared (Fig. 4), *CnCHS* alone (1 gene), followed by stepwise addition of *CnF3’H* (2 genes), *CnFLS1* (3 genes), *CnUFGT14* (MT370519.1) (4 genes), and finally *SlMYB12* (5 genes).

Transient expression of *CnCHS* in *N. benthamiana* leaves resulted in the detection of its immediate product, naringenin chalcone, alongside naringenin 7-*O*-glucoside (Na7G) and DHK (Fig. 4b). This confirms that *CnCHS* successfully established the flavonoid backbone upon heterologous expression. Notably, a portion of the precursor naringenin chalcone was further processed into downstream products by the endogenous *N. benthamiana* flavonoid biosynthetic machinery. Infiltrating leaves with *CnCHS* and *CnF3’H* led to the disappearance of early pathway intermediates (naringenin chalcone, Na7G, DHK) (Fig. 4c), replaced by the 3′-hydroxylated products DHQ and dihydroquercetin 3-glucoside (DHQ3G). This shift confirms the catalytic activity of CnF3’H in the flavonoid biosynthetic pathway.

The addition of *CnFLS1* resulted in the emergence of flavonol derivatives like kaempferol 3-glucoside (Ka3G), quercetin 3-glucoside (Qu3G), and rutin, suggesting its ability to convert flavanones to flavonols (Fig. 4d). Notably, the absence of non-glycosylated flavonols points to a robust endogenous glycosylation system in *N. benthamiana*, readily modifying newly produced flavonols. Additionally, *CnUFGT14* further enhanced the accumulation of glycosylated products like Na7G, DHQ3G, Ka3G, and Qu3G, solidifying its role as a flavonoid glycosylation enzyme, potentially with broader substrate specificity (Fig. 4e). Upon introducing *SlMYB12*, flavonoid production was significantly enhanced. But the increase in flavanones and flavonols was disproportionate. For instance, flavanones Na7G and DHQ3G exhibited <4-fold increase, from 0.50 and 0.27 μg/g to 1.71 and 0.88 μg/g, respectively, while flavonols rutin and Qu3G surged by >10-fold, from 1.25 and 2.10 μg/g to 40.02 and 19.64 μg/g (Fig. 4e, f). This disproportionate boost likely arises from two mechanisms. First, increased early pathway intermediates, such as NaCha, provided ample substrate for efficient conversion into flavonol derivatives by *CnF3’H, CnFLS1*, and *CnUFGT14*. Second, *SlMYB12* may have preferentially activated the expression of endogenous flavonol biosynthetic genes compared to other steps.

In summary, the characteristic flavonol-rich profile of *C. nitidissima* petals was successfully recapitulated in *N. benthamiana* through the transient expression system. These findings align with our hypothesis that *CnCHS, CnF3’H, CnFLS1, CnUFGT14*, and *MYB12* are integral to the biosynthesis of flavonol pigments in *C. nitidissima* petals. Furthermore, this opens exciting possibilities for utilizing these genes to reconstruct *C. nitidissima* flavonols in other horticultural crops, potentially enhancing their quality.

### Pathway reconstruction in tomato enhanced the accumulation of *C. nitidissima* flavonols

Tomato fruits, while replete with lipophilic carotenoids, contain only limited quantities of hydrophilic flavonoids, thus narrowing their nutritional spectrum [16, 23]. In this study, we sought to determine whether the expression of the five core *C. nitidissima* genes would be sufficient for the heterologous reconstruction of its flavonols in a stable transgenic tomato plant and to develop novel tomato germplasm enriched with flavonols derived from the *Camellia* species.

**Figure 5:**
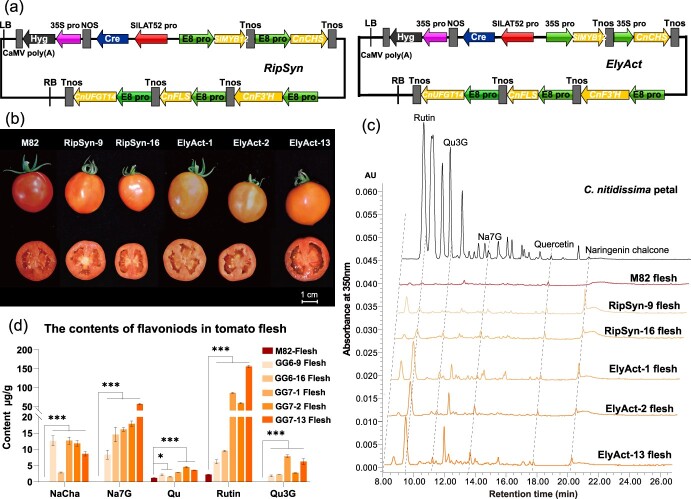
Reconstruction of the *C. nitidissima* flavonols biosynthetic pathway in cultivated tomato fruit flesh. (a) Schematics of the multigene expression vectors assembled via the TGS II system are presented. The RipSyn vector features all genes under the regulation of the *E8* promoter, while in the ElyAct vector, *SlMYB12* and *CnCHS* are governed by the 35S promoter, with the remaining genes controlled by the *E8* promoter. (b) Photographic depiction of fruits from stable transgenic tomatoes with *S. lycopersicum* M82 as the parent. Fruits from the transgenic RipSyn and ElyAct lines exhibit a more pronounced yellow hue compared to the non-transgenic M82 counterpart. The scale bars in the figure represent a length of 1 cm. (c) HPLC chromatograms display the flavonoid profiles extracted from *C. nitidissima* petals and tomato fruit flesh of the transgenic lines. In comparison to the parental M82 line, the five assessed transgenic lines reveal increased flavonoid levels, including rutin, Qu3G, Na7G, quercetin, and naringenin chalcone, which co-elute with the corresponding compounds from *C. nitidissima* petals. (d) Quantitative analysis of select flavonoids in tomato flesh, including naringenin chalcone, Na7G, quercetin, rutin, and Qu3G. Notably, the ElyAct lines produce a greater flavonol content than the RipSyn lines, with rutin and Qu3G being particularly abundant. The presented data are mean ± SEM from three biological replicates. Statistical significance between control and treated groups was assessed using an unpaired *t*-test. Significance levels are indicated by asterisks: ^*^P < 0.05, ^**^P < 0.01, ^***^P < 0.001.

We selected the cultivated tomato variety *Solanum lycopersicum* M82 as our model for *C. nitidissima* flavonol pathway engineering. Before our plant transformation experiments, we first assessed the native flavonoid biosynthesis in M82 tomato fruits (Supplementary Fig. S11a). The flavonoid content was quantified in both the flesh and peel of M82 fruits at the ripening stage. The results revealed that the flesh of M82 fruits contained negligible levels of flavonoids, while the peel presented a higher, albeit still limited, variety of these compounds (Supplementary Fig. S11b, d). Publicly available data [24] corroborated these findings, showing low expression of flavonoid biosynthesis genes across the fruit, except for *SlCHS1*, *SlCHS2*, and *SlFLS*, which were notably active in the ripening peel (Supplementary Fig. S11c). Based on these findings, we designed two sets of promoter-gene constructs using the multigene transformation system (Fig. 5a). The first, termed the Ripening-Synchronized (RipSyn) design, utilized the fruit-ripening-specific E8 promoter to drive all five genes for coordinated expression during ripening. The second design, called Early-Activation (ElyAct), combined the constitutive 35S promoter for *SlMYB12* and *CnCHS* with the E8 promoter for *CnF3’H*, *CnFLS1*, and *CnUFGT1*, aiming to trigger early expression of flavonoid-related genes and enable the accumulation of pathway intermediates prior to ripening for effective flavonol biosynthesis.

Multiple independent transformation lines for each of the two constructs—RipSyn and ElyAct—were successfully generated. From real-time quantitative PCR (RT-qPCR) analysis, two lines from the RipSyn construct and three lines from the ElyAct construct, all exhibiting detectable expression levels of the genes *SlMYB12, CnCHS, CnF3’H, CnFLS1*, and *CnUFGT1* (Supplementary Fig. S12), were chosen for further flavonoid quantification. HPLC analysis indicated that the flavonol pigments characteristic of *C. nitidissima* were successfully synthesized in the tomato flesh, imparting a yellowish hue to the fruit (Fig. 5b). Compared to the non-transgenic M82 control fruits, all selected transgenic lines demonstrated elevated levels of flavonoids, such as rutin, Qu3G, Na7G, quercetin, and naringenin chalcone (Fig. 5d). These compounds co-eluted with those in the *C. nitidissima* petal extract, as shown in Fig. 5c. To determine whether the golden-yellow hue of the transgenic tomato fruits is due to elevated flavonol levels, we analyzed the lycopene and β-carotene content (Supplementary Fig. S13), alongside flavonols in the flesh of various transgenic lines. Subsequently, we reproduced the fruit coloration *in vitro* by blending standard solutions of flavonols and carotenoids in ratios reflecting their concentrations in 0.5 g of fresh fruit, as shown in Supplementary Fig. S13.

Interestingly, the flavonoid profile suggested that the ElyAct lines produced a higher overall flavonoid content compared to the RipSyn lines, especially certain flavonol compounds (specifically rutin and Qu3G). This finding supports the hypothesis that allowing for the early accumulation of pathway intermediates before the onset of ripening can enhance flavonol accumulation. This conclusion aligns with the results obtained from transient expression assays in *N. benthamiana* (Fig. 4d).

Further examination revealed that the ElyAct transgenic lines also contained additional compounds that co-eluted with the *C. nitidissima* petal extract but were not included in the set of flavonoid standards available for the analysis (Fig. 5c). These unidentified compounds would require further investigation using liquid chromatography–mass spectrometry (LC–MS) to determine their structures and further understand their contribution to the flavonoid profile of the transgenic tomatoes.

### The fruit flesh of the ElyAct tomato line accumulated diverse *Camellia*-derived flavonols, alongside an augmented antioxidant capacity

To delve deeper into the flavonoid metabolic alterations within the fruit of transgenic tomatoes harboring the *C. nitidissima* flavonol pathway, we further analyzed the ElyAct line (ElyAct-1) that demonstrated the highest accumulation of flavonoids using LC–MS. The data corroborated the presence of flavonoids previously identified by HPLC (Supplementary Fig. S14).

**Figure 6:**
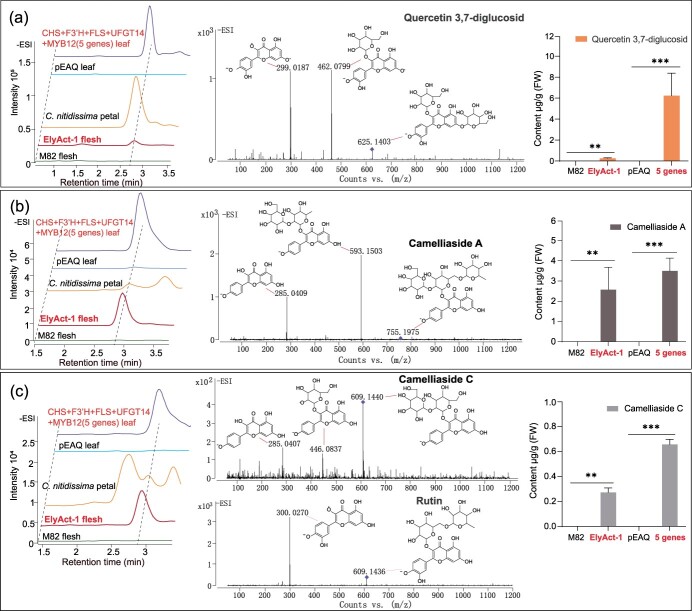
LC–MS analysis of *Camellia*-derived flavonols in the fruit flesh of the tomato ElyAct line. Comprehensive LC–MS analysis of the ElyAct tomato line fruit flesh uncovered a spectrum of *Camellia*-specific flavonols. The representative chromatograms and electrospray ionization (ESI) mass spectra were shown for selected flavonols: quercetin 3,7-diglucoside (a), camelliaside A (b), and camelliaside C (c). Notably, camelliaside C shares its molecular weight with rutin but is differentiated by its unique fragment ions. The quantification for each flavonol is presented to the left in each panel. Mass spectrometry was performed in negative-ion mode at a collision energy of 35 V. The presented data are mean ± SEM from three biological replicates. Statistical significance between control and treated groups was determined by an unpaired *t*-test, with asterisks denoting the level of significance (^*^P < 0.05, ^**^P < 0.01, ^***^P < 0.001).

In addition to the recognized metabolites, we probed the MS/MS spectra for unique *m/z* values indicative of flavonoid fragments, aiming to uncover novel flavonoid derivatives. We detected a quercetin-3,7-diglucoside (Qu3,7G) derivative in ElyAct tomato fruits, with the MS/MS fragmentation indicative of a di-glycosylated quercetin structure (Fig. 6a), mirroring the quercetin di-glycoside found in *C. nitidissima* petals, as evidenced by identical *m/z* values and retention times (Fig. 6a). Remarkably, we identified two additional flavonol derivatives in the ElyAct tomato fruits, which are the same in molecular weight and MS/MS fragmentation patterns with camelliaside A and camelliaside C, both naturally occurring in *C. nitidissima* petals (Fig. 6b, c). Both flavonols feature a kaempferol backbone decorated with either a trisaccharide (camelliaside A) or a disaccharide (camelliaside C) moiety at the 3-position. These compounds, primarily found in the flowers and seeds of Theaceae family, including *Camellia* species, are known for their potent antioxidant and anti-inflammatory properties [4, 25, 26]. Reexamination of *N. benthamiana* leaves transiently expressing the full suite of five genes also confirmed the presence of these two compounds (Fig. 6b, c). Collectively, these findings robustly demonstrate that a spectrum of *Camellia*-specific flavonol compounds, such as camelliaside A and C, can be successfully synthesized in tomato fruits through strategic expression of *C. nitidissima*-derived transgenes.

These polyglycosides of flavonoids can have antioxidant properties [27, 28]. Given this, we hypothesized that the reconstructed *C. nitidissima* flavonols in tomato, particularly those with the quercetin core, would enhance the fruit antioxidant activity. We tested this hypothesis by measuring the total oxidation capacity (TOC) and DPPH free radical scavenging capacity of fruits from the ElyAct-1 line (high flavonoid) and a RipSyn-9 line (medium flavonoid), compared to control M82 fruits. As expected, the ElyAct-1 line exhibited significantly higher TOC than both the RipSyn line-9 and M82, with the RipSyn-9 line also showing increased activity compared to M82 (Supplementary Fig. S15a). Similarly, both ElyAct-1 and RipSyn-9 lines displayed enhanced DPPH scavenging capacity compared to M82 (Supplementary Fig. S15b). These results firmly link the elevated flavonol levels in the engineered tomatoes with their increased antioxidant activity. This successful reconstruction of *C. nitidissima* flavonol pathways in tomato opens exciting possibilities for creating novel tomato germplasm with enhanced nutritional value and potentially extended shelf life.

## Discussion

### The determination of flower pigmentation in *C. nitidissima* is a complex process governed by the interplay of multiple genes

Analysis of yellow *Camellia* petals across 23 species revealed that quercetin flavonols, such as Qu3G and Qu7G, are the predominant pigments in golden *Camellia* flowers, corroborating major previous findings [4, 29]. Flavonols are a common subclass of flavonoids characterized by a C2-C3 double bond, a carbonyl group at C4, and a closed C ring structure [28]. These naturally occurring compounds are found in many fruits, vegetables, and beverages, and are known for their strong antioxidant properties, offering various health benefits. Based on structural differences, common flavonols can be categorized into quercetin, kaempferol, myricetin, and isorhamnetin [30]. FLS is pivotal in flavonol biosynthesis [20, 31] and has been demonstrated to play a critical role in determining yellow versus white flower and seed coat colors in Arabidopsis [32], as well as influencing the floral pigmentation in chrysanthemum, tree peony, and other species [33, 34]. FLS exhibits dual enzymatic functions, catalyzing either the conversion of dihydrokaempferol to kaempferol or the synthesis of quercetin from dihydroquercetin [20, 35]. Quercetin, which contains an additional hydroxyl group compared to kaempferol, boasts a stronger reducing capability and displays superior antioxidant properties [36, 37]. We determined the kinetic parameters of CnFLS1 and found that its affinity for DHQ is significantly higher than for DHK, indicating a greater substrate preference for DHQ (Fig. 3). Furthermore, compared to FLS enzymes of various other plants such as tomato, Arabidopsis, and tea, CnFLS1 exhibits significantly enhanced catalytic efficiency for DHQ (Table 1). With the same substrate, CnFLS1 can catalyze the production of more quercetin. Therefore, we conclude that FLS predominantly catalyzes the formation of quercetin derivatives, highlighting its crucial role in quercetin flavonol biosynthesis in *C. nitidissima* petals.

However, the sole expression of *CnFLS1* in tobacco did not yield a significant yellow coloration in flowers, suggesting that FLS does not act in isolation [8]; in many plants, FLS works in concert with an array of other enzymes. The interplay and equilibrium between FLS and DFR are known to modulate the ratio of flavonols to anthocyanins, consequently influencing flower color [11, 12]. Similarly, the coordination between FLS and F3’H leads to the production of distinct flavonol profiles [38]. CHS provides precursors for downstream flavonol synthesis and is a pivotal enzyme within the flavonoid pathway, but the overexpression of *CnCHS* in *C. nitidissima* alone could not significantly increase the flavonol contents [7], indicating the necessity of coexpression with *CnFLS1*. Furthermore, the quercetin produced by FLS is prone to degradation and thus requires stabilization through glucosylation by UDP-glucosyltransferase (UFGT). In *N. benthamiana* transformation experiments, we found that the synthesis of quercetin-3-glucoside and related compounds occurred only when FLS, CHS, and F3’H were coexpressed. And when UFGT was also coexpressed, the Qu3G content significantly increased (Fig. 4). Taken together, our study uncovered a complex coexpression network involving *FLS* and genes such as *CHS, F3’H*, and *UFGT*, which collectively orchestrate *C. nitidissima* flower color formation.

### Awakening silent metabolism: unveiling the hidden metabolic potential through metabolic engineering

The intricate metabolic pathways within plants often contain hidden threads of ‘silent’ or ‘underground’ metabolism–biochemical routes that, under normal circumstances, are inactive or operate at low fluxes that they remain silent [39, 40]. However, when the organism is subjected to genetic modification, such as the introduction of new biosynthetic genes, these silent pathways can become active, leading to the production of novel metabolites. This unexpected activation of silent metabolism often occurs in metabolic engineering projects, where the introduction of foreign enzymes can lead to the utilization of endogenous substrates by the engineered pathways, resulting in the formation of unanticipated products [41].

In this study, the transformation of *C. nitidissima* flavonol biosynthesis genes into *N. benthamiana* and tomato plants reveals a remarkable example of this phenomenon, highlighting the potential of silent metabolism to expand the outcomes of metabolic reconstruction. The introduction of the *C. nitidissima* genes *CnF3’H* and *CnFLS1* into *N. benthamiana* led to the production not only of flavonol core structure but also glycosylated flavonols such as Ka3G, Qu3G, and rutin (Fig. 4). These outcomes underscore the inherent but previously uncharacterized ability to glycosylate flavonols of the hosts. Similarly, the production of camelliaside A and C in transgenic tomatoes is indicative of an endogenous glycosylation system in the host that was previously silent due to the lack of flavonol substrates. Once the metabolic engineering provided sufficient substrate, these silent glycosylases were able to act, generating the *Camellia*-specific compounds (Fig. 6). The activation of silent metabolic pathways is not unique to this study. For instance, in the engineering of petunia plants with linalool, unexpected side products S-linalyl-β-D-glucopyranoside arose due to the endogenous enzymes in the petunia acting on the newly introduced intermediates [42]. Another example is introducing the *Ocimum basilicum* geraniol synthase gene in ripening tomatoes led to a cascade of monoterpenes, including unexpected compounds like limonene, myrcene, and various ocimene isomers, which efficiently increased the diversity of tomato flavor compounds [43]. These cases illustrate how silent metabolism can broaden the diversity of compounds produced through metabolic engineering, sometimes yielding products with enhanced or novel properties.

Recognizing and leveraging silent metabolism in metabolic engineering has profound implications. It offers a means to access the full metabolic potential of an organism, opening up new avenues for the production of valuable compounds. Moreover, it can lead to the discovery of novel pathways and enzymatic activities that can be harnessed for synthetic biology applications. In the context of this study, the production of camelliaside A and C in tomatoes not only enriches the fruit flavonoid profile but also potentially enhances its nutritional and health-promoting properties, given the antioxidant and anti-inflammatory attributes of these compounds. However, it is crucial to acknowledge the challenges associated with harnessing silent metabolism, such as the need for better predictive tools and a deeper understanding of the potential unintended consequences.

### Tailoring plant synthetic biology for enhanced nutritional quality of tomato fruit

Tomatoes, as a vital horticultural crop, have diverse uses. They are consumed both cooked and fresh, necessitating enhanced nutritional quality. While there is significant scope to enrich the nutritional composition of tomatoes, particularly with flavonols as investigated in this study [15, 17, 44], synthesizing such compounds can be intricate, and conventional transgenic techniques may fall short. Hence, integrating synthetic biology concepts with multigene transformation approaches is crucial for more efficient enhancement.

Recently, synthetic biology has emerged as a prominent field, with numerous laboratories employing its methods to produce specialized compounds or pharmaceuticals, including flavonoids. However, most of these efforts utilize microorganisms, with plants being less commonly employed [45]. Leveraging plant synthetic biology can harness plant-derived carbon sources, offering potential for improving nutritional content and value in edible crops, with implications for health products and pharmaceuticals [28, 46]. Moreover, horticultural infrastructure allows for rapid, cost-effective scaling, leading to a growing interest in plant synthetic biology, with implementations in crops like rice and maize [47, 48]. Yet, tomato synthetic biology remains underexplored.

In this research, we adapted a multigene transformation system previously validated in rice for enhancing anthocyanins and other beneficial compounds [48, 49]. We tailored this system for the tomato, optimizing it for multigene assembly in ripening tomato fruit. Our refined system not only establishes a foundation for tomato synthetic biology but also serves as a practical tool for flavonol synthesis, promising broader applications to elevate the nutritional value of tomatoes.

## Materials and methods

### Plant materials and growth conditions

A collection of 23 golden *Camellia* species was obtained from the National Camellia Germplasm Resource Bank (E 108°20′53“, N 22°49’11”, 75 m above sea level) in Nanning, Guangxi Province, China. Specimens were documented photographically and their color hues quantified with an NF555 colorimeter (Nippon Denshoku Industries Co., Ltd., Japan). The petals of non-yellow camellia varieties—*Camellia japonica* ‘Yudan’ (white), *C. japonica* ‘Jiuzhonghua’ (pink), *C. japonica* ‘Naidong’ (red), *C. japonica* ‘Chidan’ (red), *C. japonica* ‘Heimofa’ (black)—were collected from the Research Institute of Subtropical Forestry, Chinese Academy of Forestry. Samples were immediately frozen in liquid nitrogen postcollection, transported to the laboratory, and preserved at −80°C for subsequent analysis.

For transformation experiments, the tomato cultivar M82 (*S. lycopersicum* L.) was propagated in tissue culture under controlled conditions (28°C temperature, 16/8 h light/dark cycle). Additionally, *N. benthamiana* was cultivated for transient gene expression assays in a plant growth chamber, maintaining a consistent environment (25°C temperature, 16/8 h light/dark cycle).

### Quantitative assay of flavonoid and carotenoids components by HPLC

For the quantification of flavonoids and carotenoids, 0.3 g of frozen tissue was extracted using 2.5 mL of a solution (methanol, water, formic acid, and trifluoroacetic acid in a ratio of 70:27:2:1). Following 24 h of dark extraction, the samples were centrifuged at 3000 *g* and 4°C for 10 min, filtered through a 0.22-μm ultrafine filter, and stored at −20°C. HPLC was conducted to quantify flavonols and anthocyanins using our previously established methods [9, 10]. An Agilent Technologies 1260 Infinity HPLC system equipped with a Waters SunFire C18 column (4.6 × 250 mm, 5 μm) was employed, with a column temperature of 30°C, a flow rate of 1.0 mL/min, and an injection volume of 10 μL. The mobile phase composition, elution process, and detection wavelengths were consistent with those detailed in earlier publications.

For carotenoids extraction, 0.6 g of the sample was processed with 3 mL of a 90:10 propyl alcohol to petroleum ether solution. After the sample was thoroughly mixed with the solution by vortex, ultrasonic treatment for 15 min at low temperatures and centrifugation at 12 000 rpm, 4°C for 15 min was performed to obtain the supernatant, which was repeated thrice and pooled. The combined extract was saponified by adding 1 M NaOH at 4°C for 6 h, then dried using a nitrogen blower and redissolved in 1 mL of acetone, and filtered through an organic microporous filter membrane (0.22 μm) into a sample vial for analysis. HPLC conditions for carotenoids were set as follows: phase A, 100% acetonitrile; phase B, 100% acetone. The elution gradient commenced with 0% phase B for the initial 10 min, increased linearly to 100% phase B from 10 to 30 min, then returned to 0% phase B from 30 to 31 min, and maintained at 0% phase B until 35 min. The flow rate was maintained at 1.0 mL/min, the column temperature at 30°C, with a detection wavelength of 450 nm.

### LC–MS analysis

To perform an untargeted analysis of flavonoids, we employed an Agilent 1290 Infinity II LC system coupled with an Agilent 6546 LC/Q-TOF mass spectrometer. The chromatographic separation was performed on a Waters ACQUITY UPLC BEH C18 Column (1.7 μm, 2.1 mm*100 mm), using solvent A (water) and solvent B (acetonitrile) as the mobile phase. The gradient profile was programmed to increase solvent B to 30% within 3 min, reach 65% at 7 min, and reach 99% at 7.1 min; then maintain this composition until 8.5 min. At 8.6 min, the system was down to 5% solvent B and held until the 10-min mark. The flow rate was set at 0.3 mL/min, the injection volume at 10 μL, and the column temperature maintained at 40°C.

For the mass spectrometric analysis, the Agilent 6545B LC/Q-TOF was performed in negative ion mode. The instrument parameters were set as follows: capillary voltage at 3500 V, nebulizer pressure at 35 psi, and fragmentation voltage at 150 V. The drying gas was maintained at a temperature of 275°C with a flow rate of 8 L/min, while the sheath gas was set to 300°C and flowed at 11 L/min. Data acquisition spanned a mass range from 50 to 1700 *m/z* and incorporated two sequential experiments with alternating collision energies: a full scan at 15 eV followed by an MS/MS scan at 35 eV.

### Transcriptome sequencing using the PacBio and Illumina platforms

For comprehensive transcriptomics analysis, the study utilized both Pacific Biosciences RS II (PacBio RS II) for third-generation sequencing and Illumina HiSeqXten platforms for second-generation RNA sequencing. The research focused on *C. nitidissima*, collecting samples from seven different plant tissues—roots, leaves, fruits, sepals, petals, stamens, and pistils—at the full flowering stage, as well as flower buds at five distinct developmental stages as illustrated in Fig. 2a. A total of 36 samples were gathered, representing 12 different materials with three biological replicates each, to ensure robust data collection for transcriptomic sequencing.

RNA extraction was performed on all 36 samples, which were then pooled together for the PacBio RS sequencing to capture full-length transcripts. In parallel, each sample was individually subjected to Illumina sequencing. The full-length cDNA required for the third-generation library construction was synthesized using SMARTer technology, which is particularly adept at generating high-quality full-length cDNA. Size selection of the full-length cDNA fragments was conducted with the BluePippin system, a preparative electrophoresis platform that allows for precise molecular weight cutoffs, facilitating the construction of a high-quality cDNA library. The PacBio RS II platform yielded 19.13 Gb of clean data, culminating in 618 999 full-length non-chimeric reads. Subsequent refinement produced 122 201 non-redundant sequences, of which 108 897 transcripts (89.11%) were successfully annotated through comparison with multiple databases, while 13 304 (10.89%) remained unannotated. Notably, 45 350 transcripts were annotated in the KEGG database (Supplementary Fig. S3a).

For the analysis of differential gene expression among the various sample groups, the study employed DESeq, a widely used statistical package for determining differential expression in digital gene expression data. The transcripts identified as differentially expressed were then annotated and subjected to enrichment analysis using Gene Ontology (GO) and KEGG databases. Additionally, the transcripts were categorized using the Cluster of Orthologous Groups (COG) and the evolutionary genealogy of genes: Non-supervised Orthologous Groups (eggNOG) databases to aid in the functional classification of the genes. The raw data has been made publicly available in the National Center for Biotechnology Information (NCBI) repository under the accession number PRJNA909942.

### Correlation analysis between DEGs and pigment contents conducted using WGCNA

Key candidate genes associated with the pigmentation of *C. nitidissima*’s yellow flowers were identified by calculating the correlation between FPKM values of differentially expressed transcripts with KEGG annotation and flavonoid content in 12 tissues, measured via HPLC, using Matlab software (details in Supplementary Data S4). Genes with significant differential expression (*P* < 0.05) underwent KEGG pathway enrichment and functional annotation to pinpoint genes involved in the flavonoid biosynthesis pathway.

WGCNA was performed using an R package (details in Supplementary Data S4). To refine the coexpression network for specificity and efficiency, genes with an average FPKM value <0.5 across tissues were excluded. The ‘pickSoftThreshold’ function in the WGCNA package determined the weight value, setting the soft-thresholding power at 10 to construct the network. Subsequently, the ‘adjacency’ and ‘hclust’ functions transformed the adjacency matrix into a Topological Overlap Matrix (TOM), followed by hierarchical clustering. The ‘cutreeDynamic’ method delineated gene clusters within the dendrogram, where each branch represented a coexpression module; modules with eigengenes correlating >0.6 were merged. A labeled heat map displaying correlations between gene modules and principal flavonoid pigments was generated. Genes with module membership weight >0.25 were selected to construct a gene coexpression network. Key hub genes and their coexpressed counterparts were visualized using Cytoscape software.

### Protein expression and enzyme kinetics analysis of FLS isoforms

To characterize the catalytic activity of CnFLS1, the ORF sequence of *CnFLS1* was cloned into the pET28b vector and transformed into *Escherichia coli* Rosetta (DE3) cells. For comparative analysis, *SlFLS* from tomato (Solyc11g013110) and *AtFLS1* from Arabidopsis (AT5G08640.1) were cloned. Transformants were selected on LB agar containing 50 mg/L kanamycin and 34 mg/L chloramphenicol, and verified using primers listed in Supplementary Table S3. Positive colonies were propagated in liquid LB at 28°C to an OD600 of 0.5–0.6, at which point protein expression was induced with 0.05 mM IPTG. Cultures were incubated at 16°C under 120 rpm shaking for ~14 h. The cells were collected by centrifugation at 5000 *g*, 4°C for 15 min, resuspended in lysis buffer (50 mM NaH_2_PO_4_, 300 mM NaCl, 10% glycerol, pH 8.0), and lysed by sonication. Cell debris was separated by centrifugation at 20 000 *g* for 30 min at 4°C. The supernatant was incubated with Ni-NTA resin in lysis buffer at 4°C for 2 h, and proteins were eluted using a gradient of imidazole. Protein purity was confirmed via SDS-PAGE and western blotting. Purified proteins were mixed with glycerol to 40% for storage at −20°C.

Enzymatic kinetic parameters for FLSs followed an established method with modifications [20, 35]. Reactions contained 100 mM Tricine (pH 7.5), 0.1 mM FeSO_4_, 10 mM ascorbate, 10 mM 2-oxoglutarate, 0.5 mg/mL catalase, 0.1 mg/mL BSA, 20 μM substrate (DHQ or DHK), and 2 μg FLS protein in a volume of 100 μL. Reactions initiated by enzyme addition proceeded at 28°C with shaking for 15 min and were quenched with 50 μL methanol. Postcentrifugation, the supernatant underwent UPLC-UV analysis with a mobile phase of solvent A (0.1% formic acid) and solvent B (0.1% formic acetonitrile). The elution gradient was from 5% B to 20% B over 1 min, then to 100% B by 7 min, holding until 8 min, returning to 5% B at 9 min, and maintaining for 1 min. The flow rate was 0.3 mL/min, injection volume was 10 μL, column temperature was maintained at 30°C, and the detection wavelength was at 350 nm.

### Transient gene expression in *N. benthamiana* leaves

To perform transient gene expression in *N. benthamiana* leaves, genes were cloned into the pEAQ vector utilizing homologous recombination, with primers detailed in Supplementary Table S3. Subsequently, the verified plasmids were transformed into *Agrobacterium tumefaciens* strain GV3101. The positive strains were cultured at 28°C and 200 rpm until reaching an OD600 of 1.0–1.2. For coinfiltration, strains harboring the target genes were mixed to equalize the final OD600 to 0.5 for each, at a 1:1 concentration ratio. The *Agrobacterium* mixture was then pelleted at 6000 *g* for 10 min, washed with 10 mM MES buffer (pH 5.6) containing 10 mM MgCl2, and centrifuged once more. The pellet was resuspended in infiltration buffer (10 mM MES pH 5.6, 10 mM MgCl_2_, 200 mM acetosyringone) to prepare the inoculum, which was incubated at room temperature for 2 h prior to infection. The prepared solution was infiltrated into the lower epidermis of leaves from 5- to 6-week-old healthy *N. benthamiana* seedlings. Four to five days post infiltration, the leaves were harvested for flavonoid extraction.

### Construction of the multi-gene expression system

The multigene stacking system (TransGene Stacking II, TGS II) [48] was utilized to assemble multigene expression vectors with slight modifications. Initially, donor vector promoters were substituted with tomato-specific promoters: the 35S promoter in the pYL322d1 vector was replaced with the E8 promoter (tomato fruit-specific expression promoter), and the PV4 promoter in the pYLMF-H vector was swapped with the LAT52 promoter (Solyc10g007270, a tomato pollen-specific expression gene). To improve the experiment’s efficiency and accelerate the process, Golden Gate cloning was employed to simplify the procedures. Once all genes were accurately integrated into the pYL322d1 vectors, primers for Golden Gate assembly were designed to concatenate all genes into a single pYL322d1 vector, as listed in Supplementary Table S3. The 20 μL Golden Gate reaction mixture consisted of 75 ng of each insert, 0.5 μL Aar I, 0.4 μL Aar I oligo, 1 μL T4 DNA Ligase, and 2 μL 10X T4 DNA ligase buffer, undergoing 45 cycles of 37°C for 1.5 min and 16°C for 1.5 min, followed by a final incubation at 37°C for 5 min and 60°C for 5 min. Subsequently, 2 μL of the reaction mix was transformed into electrocompetent T10 cells. The next phase involved CRE-mediated recombination, assembling all genes into the plant expression vector pYLTAC380GW. Finally, Gateway BP cloning was used to insert a hygromycin resistance marker. 

### Genetic transformation of tomato and evaluation of transgenic lines

Cotyledons of the cultivated tomato *S. lycopersicum* M82 were employed for transformation. Briefly, M82 tomato seeds were sterilized and germinated under sterile conditions until cotyledons appeared. These cotyledons were excised and dark-incubated for 1 day before transformation. *Agrobacterium tumefaciens* strain GV3101 harboring the final vector was cultured in LB medium supplemented with 50 mg/mL kanamycin and 50 mg/mL rifampicin until an OD600 of 0.8–1.0 was reached. The cells were resuspended in a suspension buffer (2.2 g MS medium, 10 g sucrose, 50 mg inositol in 500 ml, and 0.4 mg/mL thiamine hydrochloride) and incubated at room temperature for 2 h. The tomato cotyledons were immersed in the bacterial suspension for 2–3 min, then placed on the cocultivation medium for 1–2 days in the dark to induce callus formation. Subcultures were performed until shoots of 2- to 3-cm length developed, followed by root induction. Rooted shoots were then planted in soil and acclimatized in the greenhouse.

Transgenic lines were confirmed via PCR using primers listed in Supplementary Table S3. Gene expression in the transgenic tomatoes was assessed in the fruit flesh at the red ripening stage. RT-qPCR was conducted for target genes, including *CnCHS*, *CnF3’H*, *CnFLS1*, *CnUFGT14*, and *SlMYB12*. Concurrently, flavonoid content in the tomato flesh was quantified through HPLC and LC–MS analyses. The total antioxidant capacity was assessed via the Ferric-Reducing Ability of Plasma (FRAP) method, following the protocol of the BC1310 assay kit (Solarbio, Beijing), with measurements carried out using a spectrophotometer. The DPPH free radical scavenging capacity was also measured using a spectrophotometer, following the instructions provided in the BC1320 assay kit (Solarbio, Beijing).

## Supplementary Material

Web_Material_uhae308

## Data Availability

The data that supports the findings of this study are available in the supplementary material of this article.
