# Supplementary material for: Elucidation of the key flavonol biosynthetic pathway in golden Camellia and its application in genetic modification of tomato fruit metabolism
Source: Hortic Res. 2024 Nov 7;12(2):uhae308. doi: 10.1093/hr/uhae308 (PMC11818005; doi:10.1093/hr/uhae308)
Supplement: Web_Material_uhae308 [file web_material_uhae308.zip › FPX-Supplementary Figures and Tables legends.docx]

**Supplement Tables and data:**

Table S1: Contents of flavonoids, anthocyanins and carotenoids in flowers of 23 species of golden *Camellia*

Table S2: Genes involved in the flavonoid biosynthetic pathway were annotated in the KEGG database that significantly associated with Qu7G, Qu3G, and TFL (p<0.05)

Table S3: Primer sequences used in the current study

Supplementary Data S1: The FPKM values of all unigenes used in the WGCNA analysis.

*After filtering out low-expression genes, 22,658 KEGG-annotated DEGs were subjected to analysis.

Supplementary Data S2: The contents of different flavonoid components in various tissues of *C. nitidissima.*

*Pigment concentrations in samples subjected to transcriptome analysis were determined via high-performance liquid chromatography (HPLC).

Supplementary Data S3: Cytoscape input edges in modules Lightcyan1 and Darkgreen (weigh > 0.25)

*Data from the Cytoscape input edges in modules Lightcyan1 and Darkgreen file generated by WGCNA analysis were further filtered to include only those with a weight value greater than 0.25, which were then used for the visualization of the co-expression gene network.

Supplementary Data S4: Scripts employed for correlation and WGCNA analyses.

*The Matlab code, denoted as a#, performs correlation analysis between differentially expressed genes (DEGs) and the levels of Qu7G and Qu3G, the yellow pigments in *Camellia* petals. The R language script, labeled as b#, is utilized for weighted gene co-expression network analysis (WGCNA).


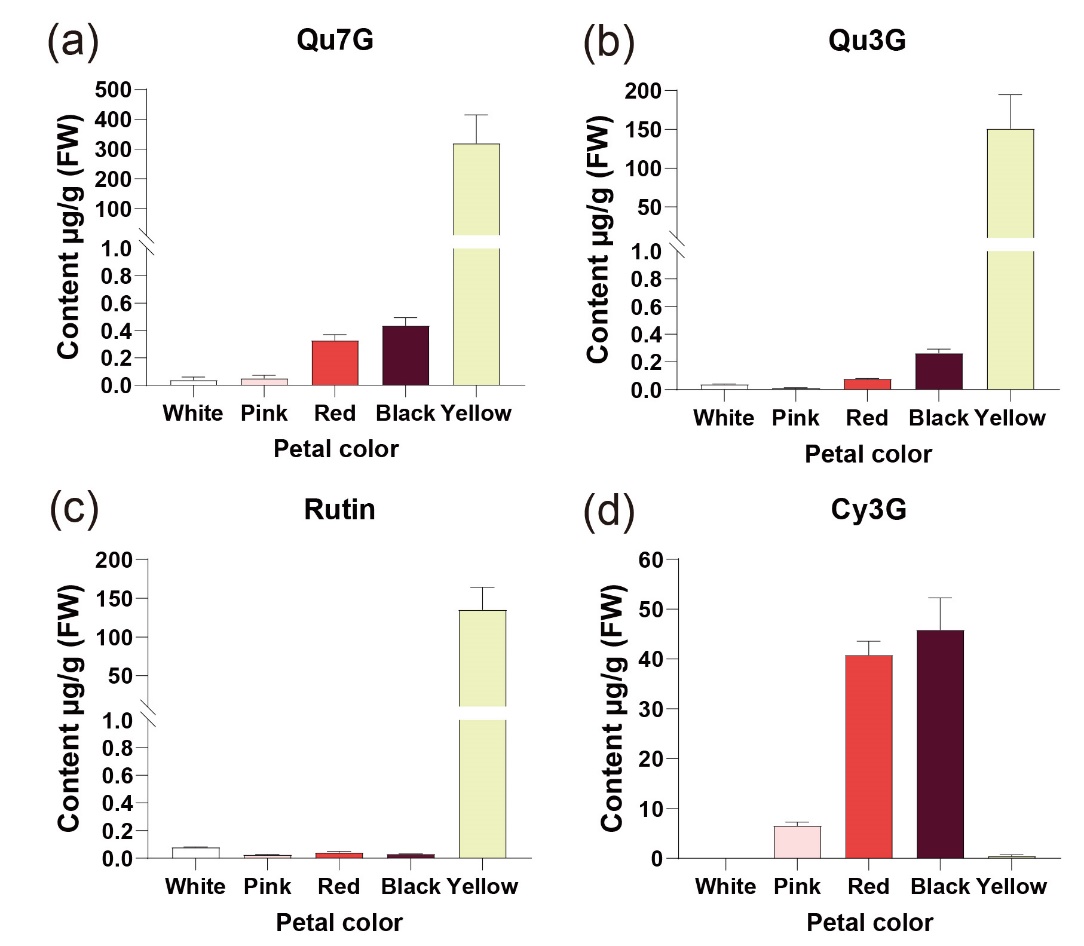


**Supplementary Figure S1. The content of several major flavonoid compounds in *Camellia* showing different flower colors.**

The content of quercetin 7-O-glucoside (Qu7G) (a), quercetin 3-O-glucoside (Qu3G) (b), rutin (c), and cyanidin 3-*O*-glucoside (Cy3G) (d) were quantified for *Camellia* flowers showing different colors – white, pink, red, black, and yellow.


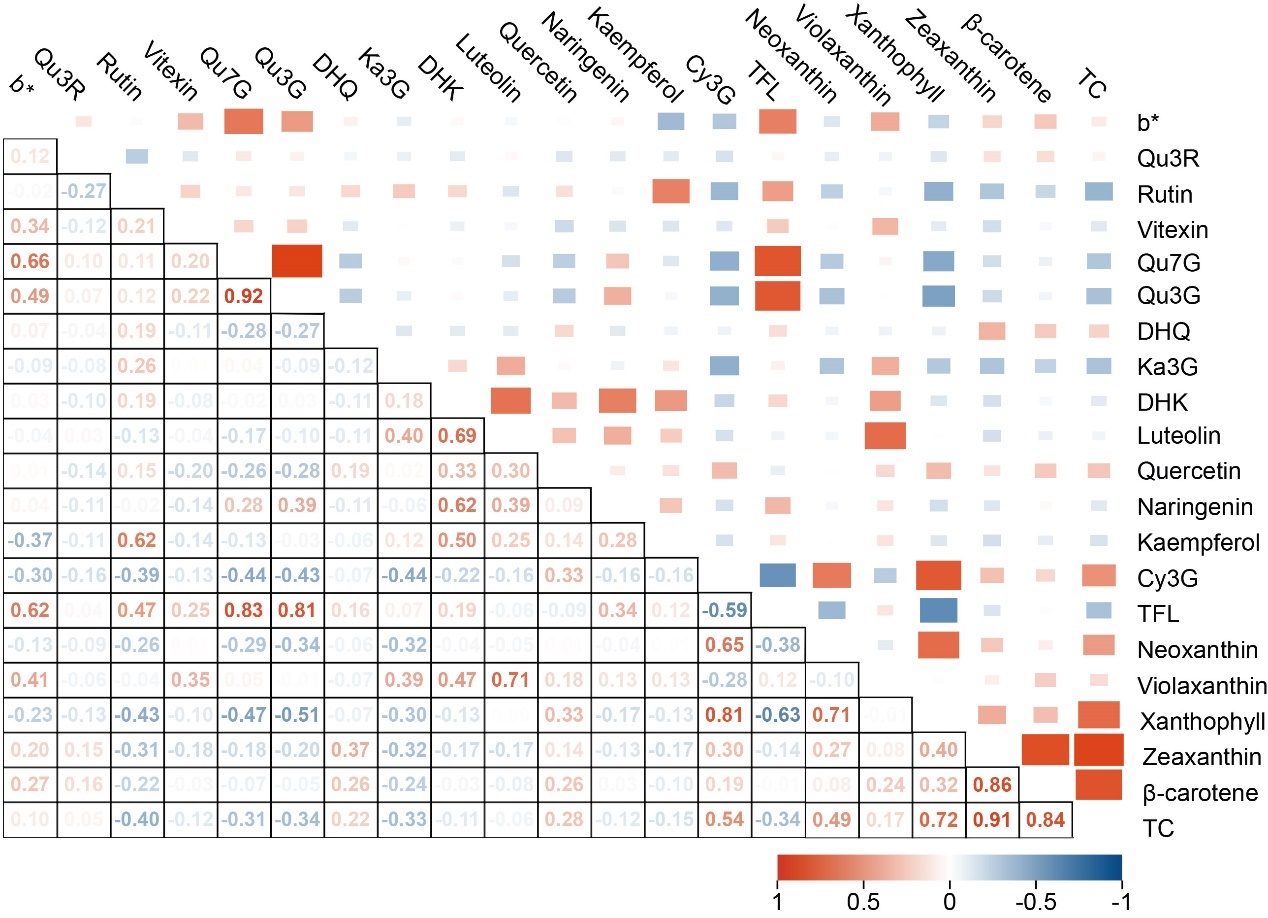


**Supplementary Figure S2. Correlation analysis between the hue b* (yellowness index) and the concentrations of various flavonoid and carotenoid pigments.**

Pearson correlation coefficients (r) were calculated for each pair within the data sets, with a 95% confidence interval. The diagram employs a color gradient where red signifies a positive correlation and blue signifies a negative correlation, with the square size denoting the correlation's magnitude. Notably, the hue b* is significantly positively correlated with quercetin 7-*O*-glucoside (Qu7G), quercetin 3-*O*-glucoside (Qu3G), and the total flavonoid content (TFL).


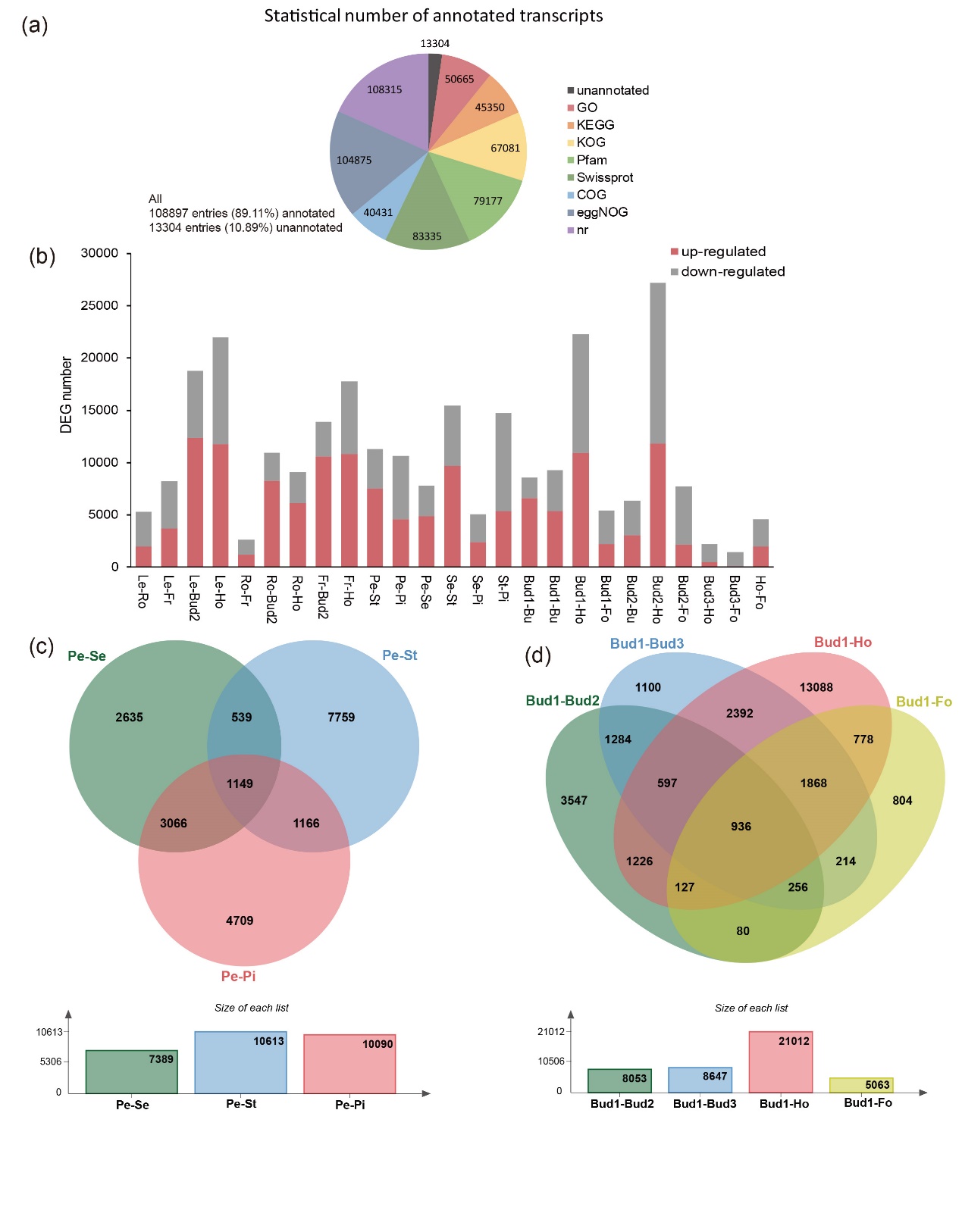


**Supplementary Figure S3. Analysis of annotated transcripts and the number of differentially expressed genes (DEGs) across tissue types.**

(a) The statistical number of annotated transcripts, with different colors representing the quantity of genes annotated in distinct databases. The gray color indicates that the 10.89% of genes that remain unannotated out of a total of 108,897 annotated genes (89.11%). (b) The number of DEGs in various tissues, where red denotes upregulated genes and gray represents downregulated ones. Sample codes are defined as follows: Le (leaves), Ro (roots), Fr (fruits), Bud1 (buds with a diameter of 10 mm), Bud2 (buds with a diameter of 20 mm), Bud3 (buds with a diameter of 30 mm), Ho (half-opened flowers), Fo (fully-opened flowers), Se (sepals), Pi (pistils), St (stamens), and Pe (petals). (b) Venn diagram illustrating the DEGs from flower tissues at five distinct developmental stages. DEGs were generated using Bud1, which has the least yellow pigmentation, as the control sample for comparison with Bud2 (green block), Bud3 (blue block), half-opened (red block), and fully opened flowers (yellow block). (d) A Venn diagram depicting the DEGs among different floral organs, with petals as the reference group and comparisons against sepals (green block), stamens (blue block), and pistils (red block).


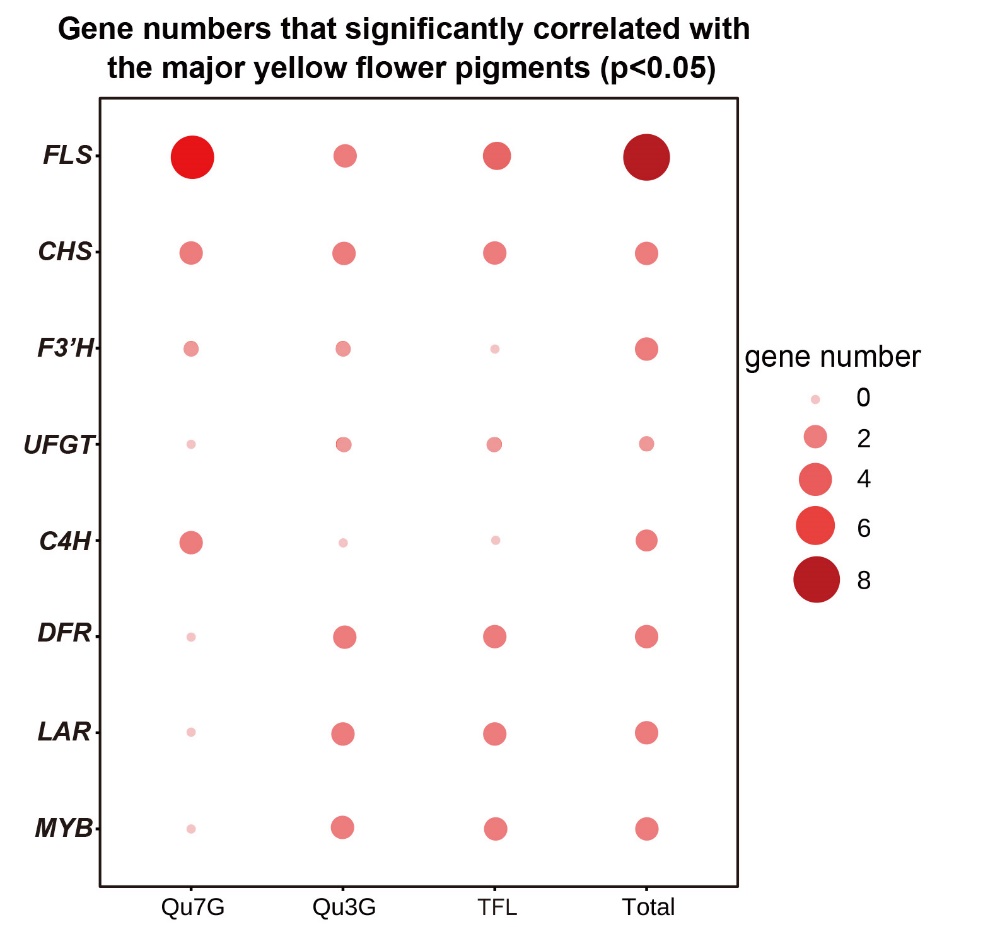


**Supplementary Figure S4. Number of unigenes involved in the flavonoid biosynthesis pathway that are significantly correlated with Qu7G and Qu3G in *C. nitidissima* petals.**

The horizontal axes represent the contents of key contributors to the unique yellow hue of *Camellia* flower, Qu7G, quercetin 7-*O*-glucoside; Qu3G, quercetin 3-*O*-glucoside and the total flavonoid content (TFL). Total represents a significant correlation with all three indicators. The ordinate shows the number of unigenes that are significantly associated with the content of pigments. The size and depth of the red circles represent the number of unigenes.


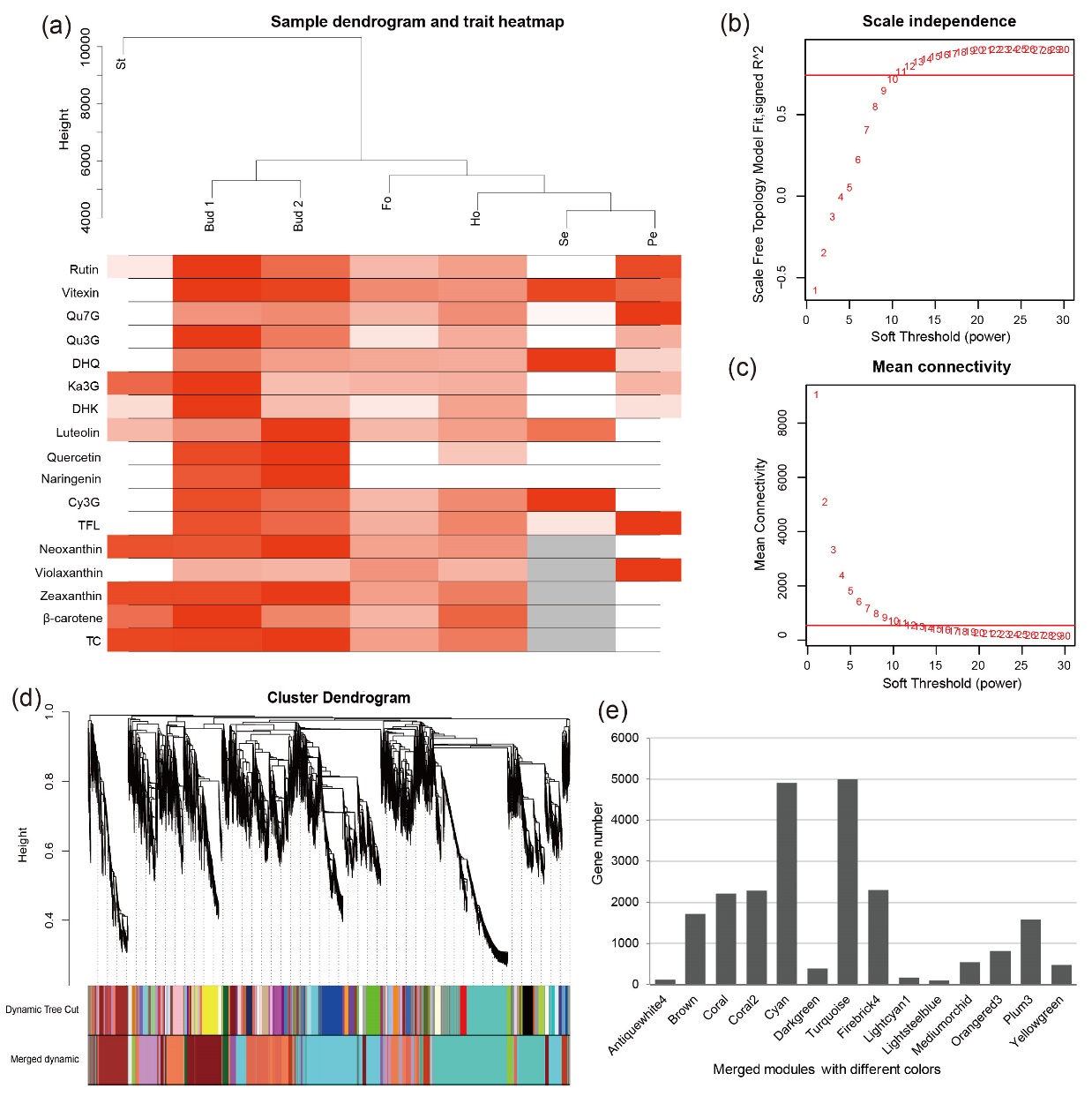


**Supplementary Figure S5. Multidimensional analysis of gene expression and pigment composition during *C. nitidissima* flower development using WGCNA analysis.**

(a) Sample dendrogram and trait heatmap. This panel presents a dendrogram resulting from hierarchical cluster analysis alongside a heatmap that delineates the association between various floral traits and pigment content across different sample types. The samples include Bud1 (buds with a 10 mm diameter), Bud2 (buds with a 20 mm diameter), Ho (half-opened flowers), Fo (fully-opened flowers), Se (sepals), St (stamens), and Pe (petals). The heatmap reflects the concentration of a spectrum of pigments, Qu7G (quercetin 7-*O*-glucoside), Qu3G (quercetin 3-*O*-glucoside), DHQ (dihydroquercetin), Ka3G (kaempferol-3-glucoside), DHK (dihydrokaempferol), Cy3G (cyanidin 3-*O*-glucoside), along with the cumulative quantities of flavonoids (TFL) and carotenoids (TC). (b) Scale independence plot. This graph depicts the relationship between the scale-free topology of the network and various soft-thresholding powers, thus illustrating the independence of the clustering from the choice of the scale parameter. (c) Mean connectivity graph. This plot showcases the average connectivity within the network modules, and how it varies across different soft-thresholding powers, demonstrating the robustness of the network connectivity. (d) Representation of the dynamic tree and gene modules. A spectrum of colors denotes distinct modules. The initial dynamic tree cut identified 90 modules, each with a unique color. After merging similar modules, 14 consolidated modules remained, each represented by a distinct color. (e) Gene distribution across modules. The bar chart in this panel displays the number of genes contained within each color-coded module, with the x-axis categorizing the modules according to their assigned colors, revealing the modular organization of gene expression patterns.


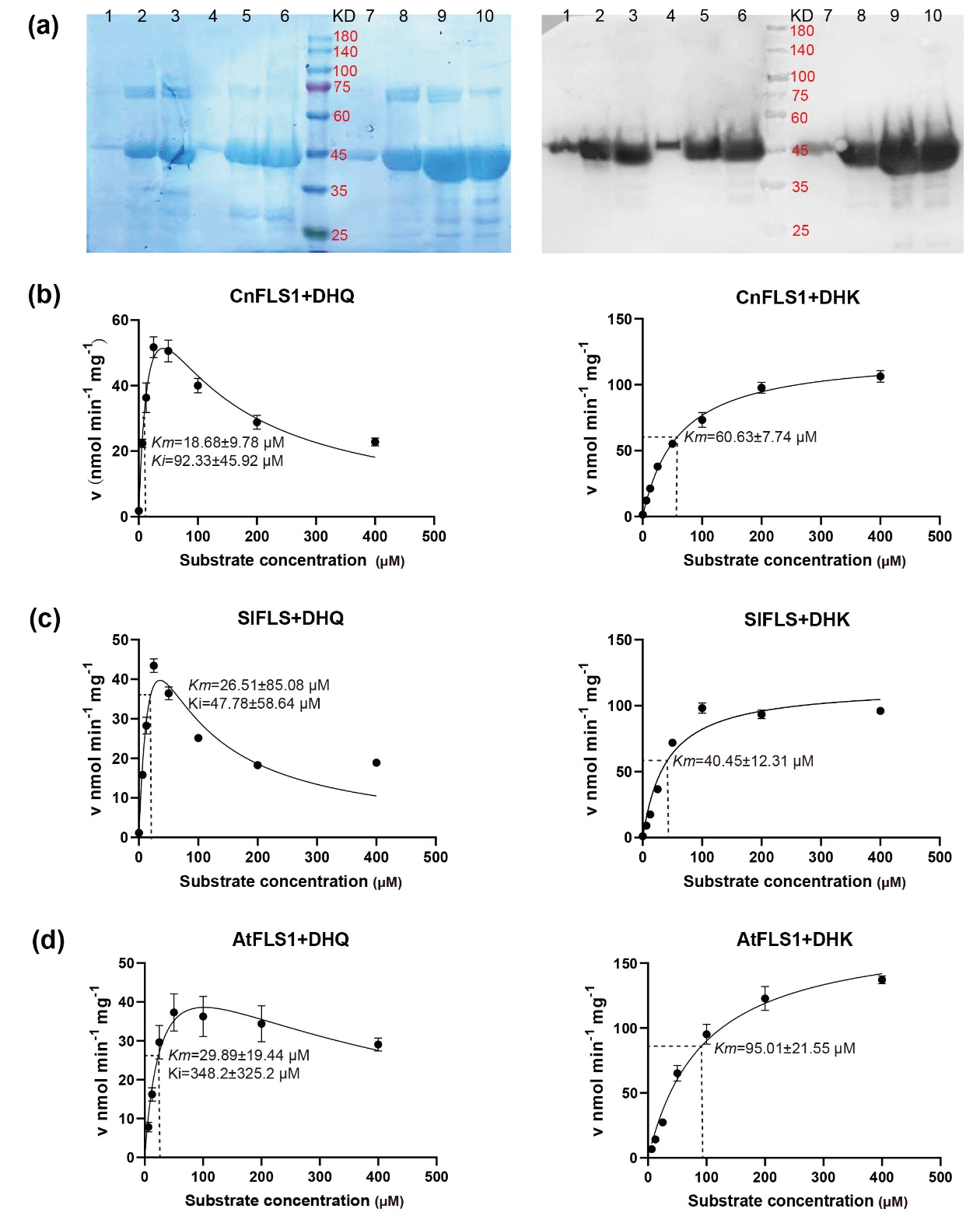


**Supplementary Figure S6. The protein expression and enzyme activity of three recombinant FLS enzymes**

(a) Protein Purification Analysis of CnFLS1, SlFLS, and AtFLS1: SDS-PAGE and Western blot assays display the purity levels of CnFLS1, SlFLS, and AtFLS1 proteins expressed in E. coli Rosetta (DE3) cells through the course of the purification process. The lanes are designated as follows: lanes 1-3 show the wash fractions of CnFLS1 with 60 mM, 150 mM, and 200 mM imidazole, respectively; lanes 4-6 display the same wash fractions for SlFLS; lanes 7-10 depict the wash fractions for AtFLS1 with 60 mM, 100 mM, 150 mM, and 200 mM imidazole, demonstrating the effectiveness of the imidazole gradient in protein elution. The Michaelis-Menten kinetics of the enzymes CnFLS1 (b), SlFLS (c), and AtFLS1(d) were analyzed with DHQ and DHK as substrates, showing the reaction velocities at various substrate concentrations. The substrate inhibition effect by DHQ at higher concentrations was observed for all three enzymes. The inhibition constant (Ki) and the Michaelis constant (Km) are calculated and listed. All kinetic data are expressed as mean ± SEM, derived from three independent biological replicates,


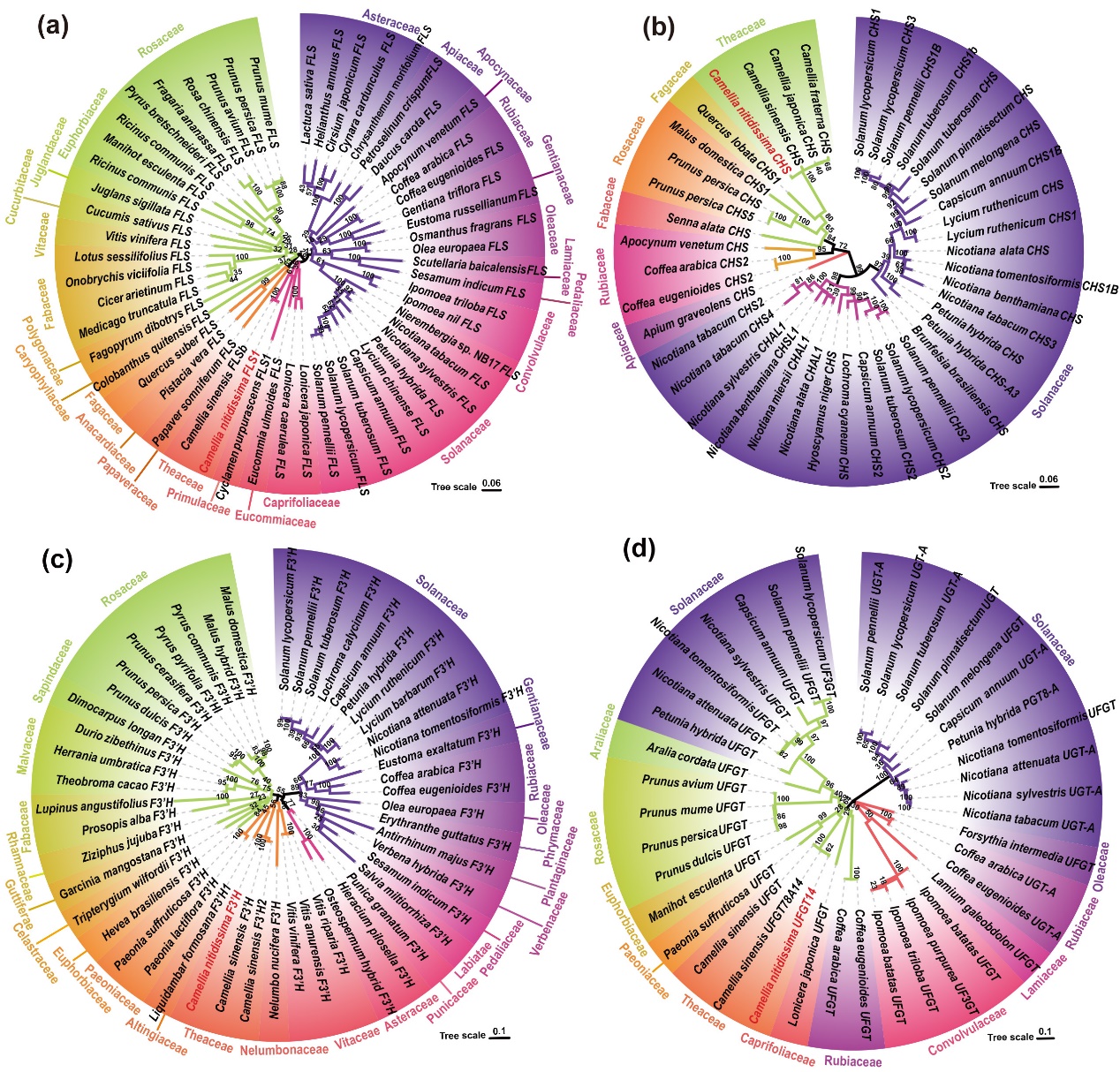


**Supplementary Figure S7. Phylogenetic relationships of structural genes involved in flavonoid biosynthesis in *C. nitidissima* alongside their homologs across diverse plant taxa.**

Phylogenetic trees displaying the genetic relationships between flavonoid biosynthesis structural genes from *C. nitidissima* and their respective homologous genes from various plant genera. Each panel represents a separate phylogenetic analysis for (a) *CnFLS1*, (b) *CnCHS*, (c) *CnF3’H*, and (d) *CnUFGT14*. The genes from different genera are color-coded for clear differentiation, with those from *C. nitidissima* distinctly highlighted in red font to emphasize their phylogenetic positioning. These trees provide insights into the evolutionary history and potential functional conservation of these genes in plant flavonoid metabolism.


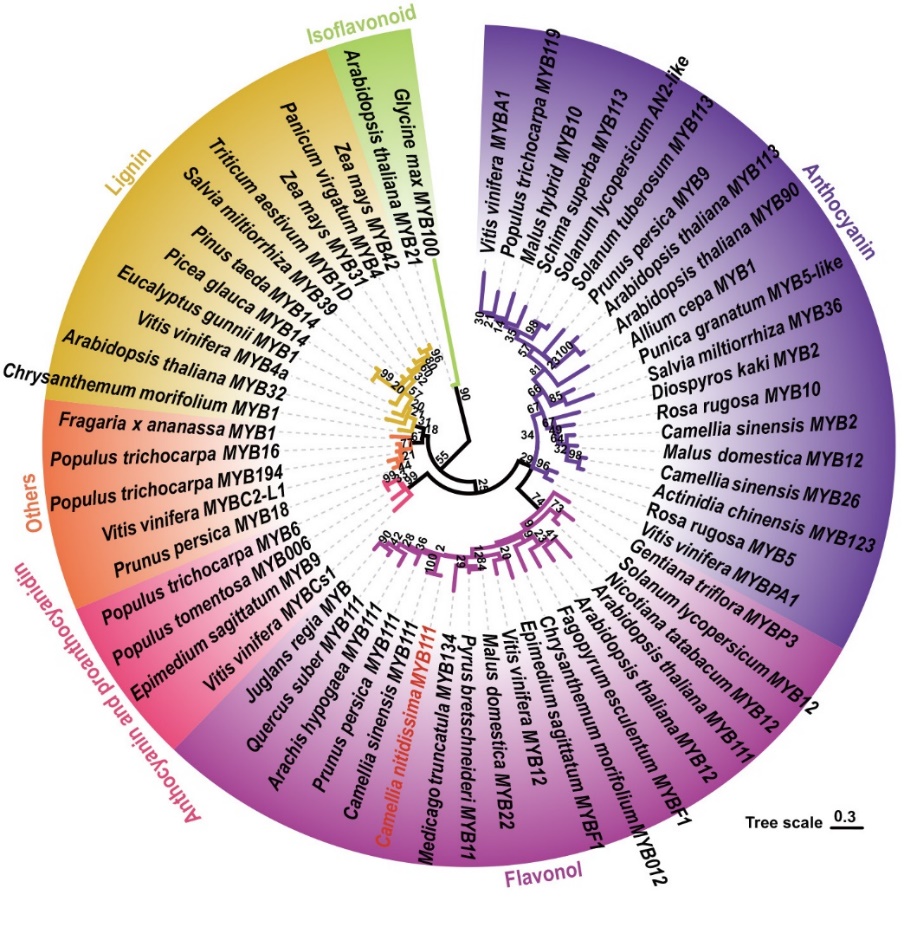


**Supplementary Figure S8. Phylogenetic snalysis of *C. nitidissima* MYB transcription factors and cross-taxa homologs in flavonoid biosynthesis regulation.**

A comprehensive phylogenetic analysis illustrating the genetic relationships between the MYB transcription factors of *C. nitidissima*, which play a crucial role in regulating flavonoid biosynthesis, and their corresponding homologous genes from a variety of plant genera. Each type of MYB, associated with the regulation of specific flavonoid subgroups, is distinctly color-coded for easy identification. The MYB transcription factor from *C. nitidissima* is highlighted in red to pinpoint its placement within the evolutionary landscape of these regulatory genes, shedding light on the conservation and divergence of flavonoid biosynthesis control mechanisms in plants.


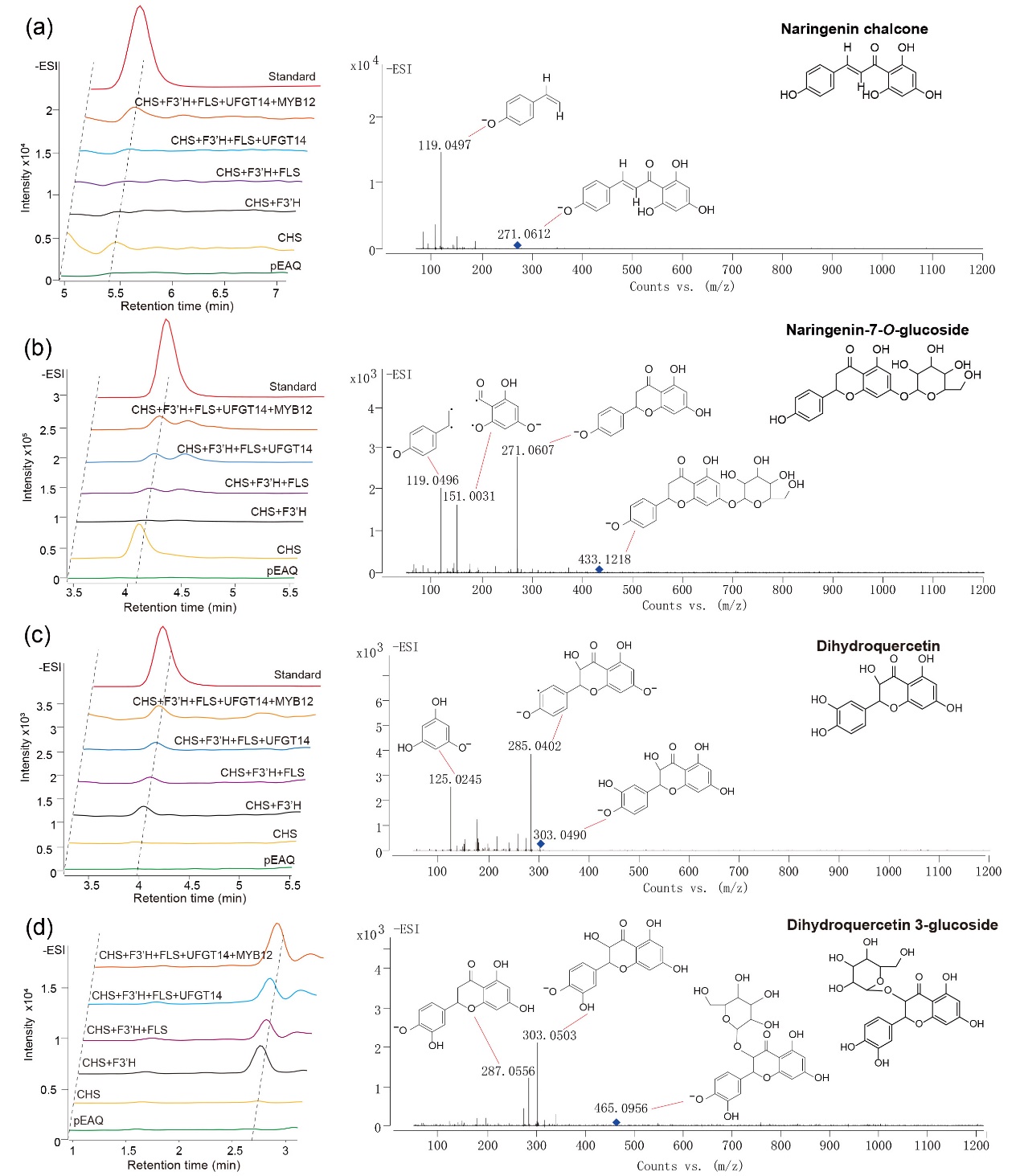


**Supplementary Figure S9. LC/MS analysis of the** **flavanones in *N. benthamiana* leaves after transient expression of genes involved in *C. nitidissima* flavonoid biosynthesis.**

Comprehensive LC/MS analysis of the *N. benthamiana* leaves uncovered a spectrum of flavanones. The representative chromatograms and electrospray ionization (ESI) mass spectra were shown for selected flavanones: naringenin-7-*O*-glucoside (a), dihydroquercetin (b), and dihydroquercetin 3-glucoside (c). Mass spectrometry was performed in negative-ion mode at a collision energy of 35 V.


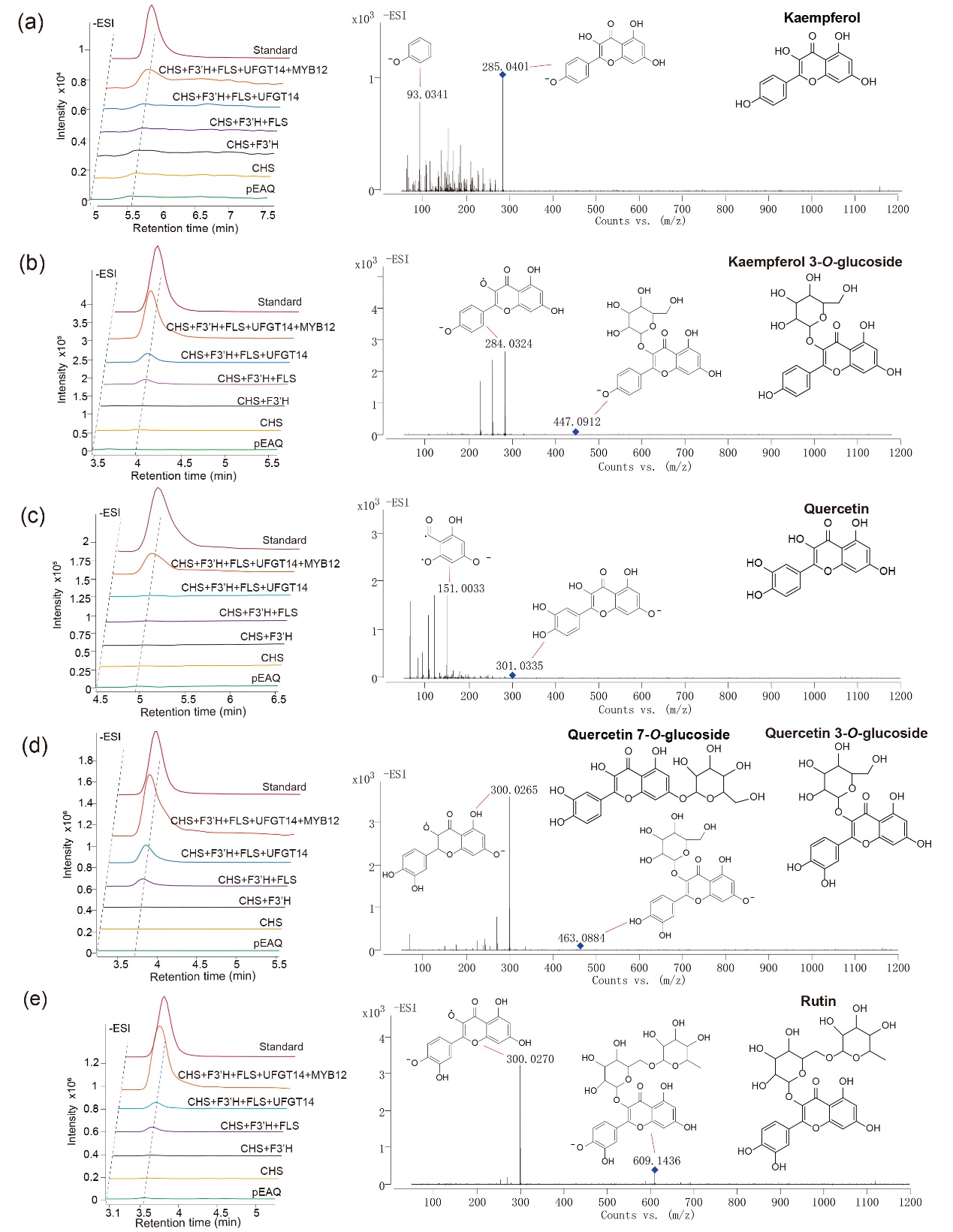


**Supplementary Figure S10. LC/MS analysis of the flavonols in *N. benthamiana* leaves after transient expression of genes involved in *C. nitidissima* flavonoid biosynthesis.**

Comprehensive LC/MS analysis revealed diverse flavonols produced in the infiltrated *N. benthamiana* leaves. The representative chromatograms and electrospray ionization (ESI) mass spectra were shown for selected flavonols: kaempferol (a), kaempferol 3-*O*-glucoside (b), quercetin (c), quercetin 7-O-glucoside and quercetin 3-*O*-glucoside (d), and rutin (e). Mass spectrometry was performed in negative-ion mode at a collision energy of 35 V.


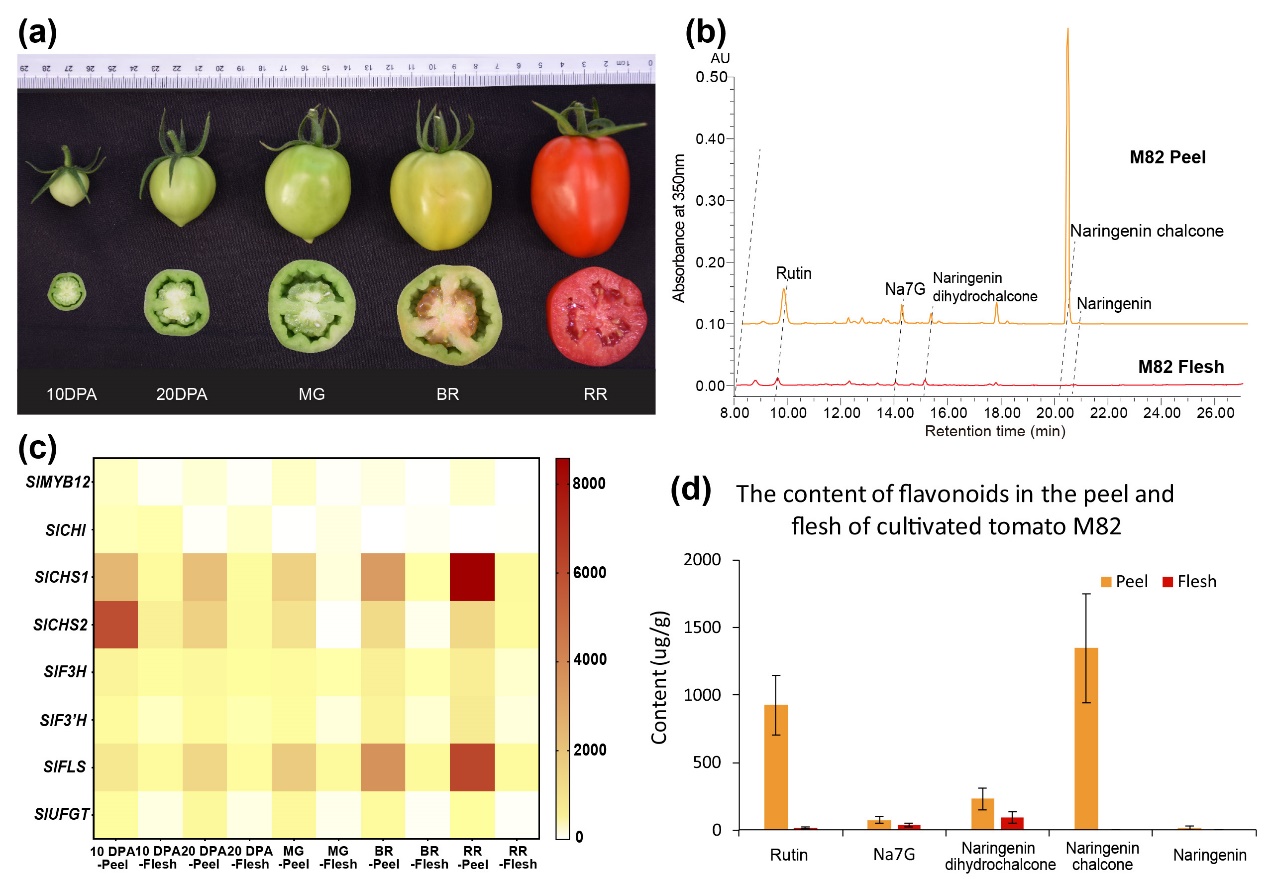


**Supplementary Figure S11. Flavonoid content quantification and gene expression analysis in fruits of cultivated tomato M82.**

(a) Photographic representation of M82 fruits at five developmental stages: 10 days post-anthesis (10DPA), 20 days post-anthesis (20DPA), mature green (MG), color breaking (BR), and red ripe (RR). (b) HPLC-detected flavonoid chromatograms in the peel and flesh of M82 tomatoes at the RR stage, with orange indicating peel content and red denoting flesh content. (c) Heatmap illustrating expression levels of pivotal genes in the flavonoid biosynthesis pathway within the peel and flesh of M82 tomatoes, data retrieved from https://tea.solgenomics.net/. (d) Comparative flavonoid contents in the peel and flesh of cultivated tomato M82, using orange for peel and red for flesh representation. Data are expressed as mean ± SEM from three independent biological replicates.


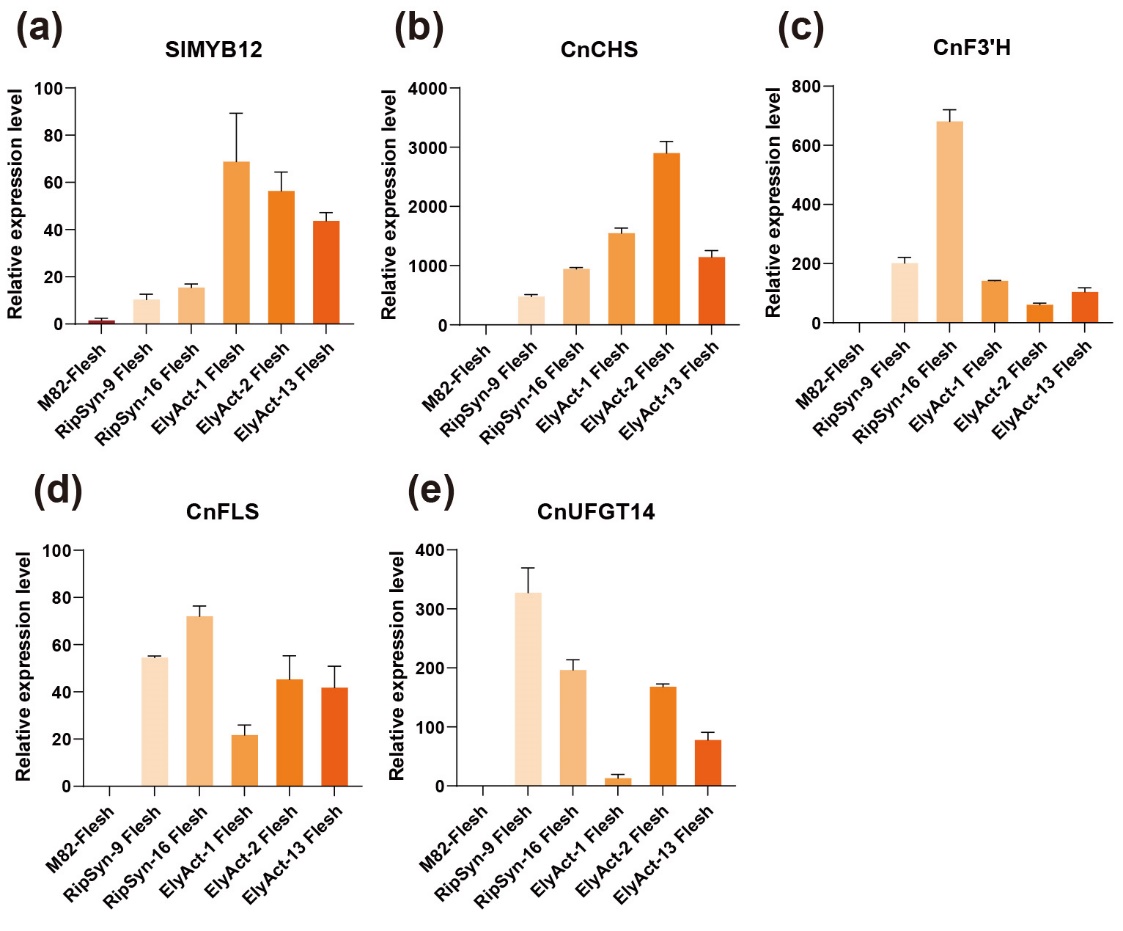


**Supplementary Figure S12. Expression levels of target genes in transgenic tomato flesh.**

The analyzed transgenes encompass *SlMYB12* (a), *CnCHS* (b), *CnF3’H* (c), *CnFLS1* (d), and *UFGT14* (e). The RipSyn-9 and RipSyn-16 lines, expressing all five target genes under the fruit-specific *E8* promoter, are compared alongside ElyAct lines—ElyAct-1, ElyAct-2, and ElyAct-13—which harbor *SlMYB12* and *CnCHS* under the 35S promoter with the remaining genes under the *E8* promoter.


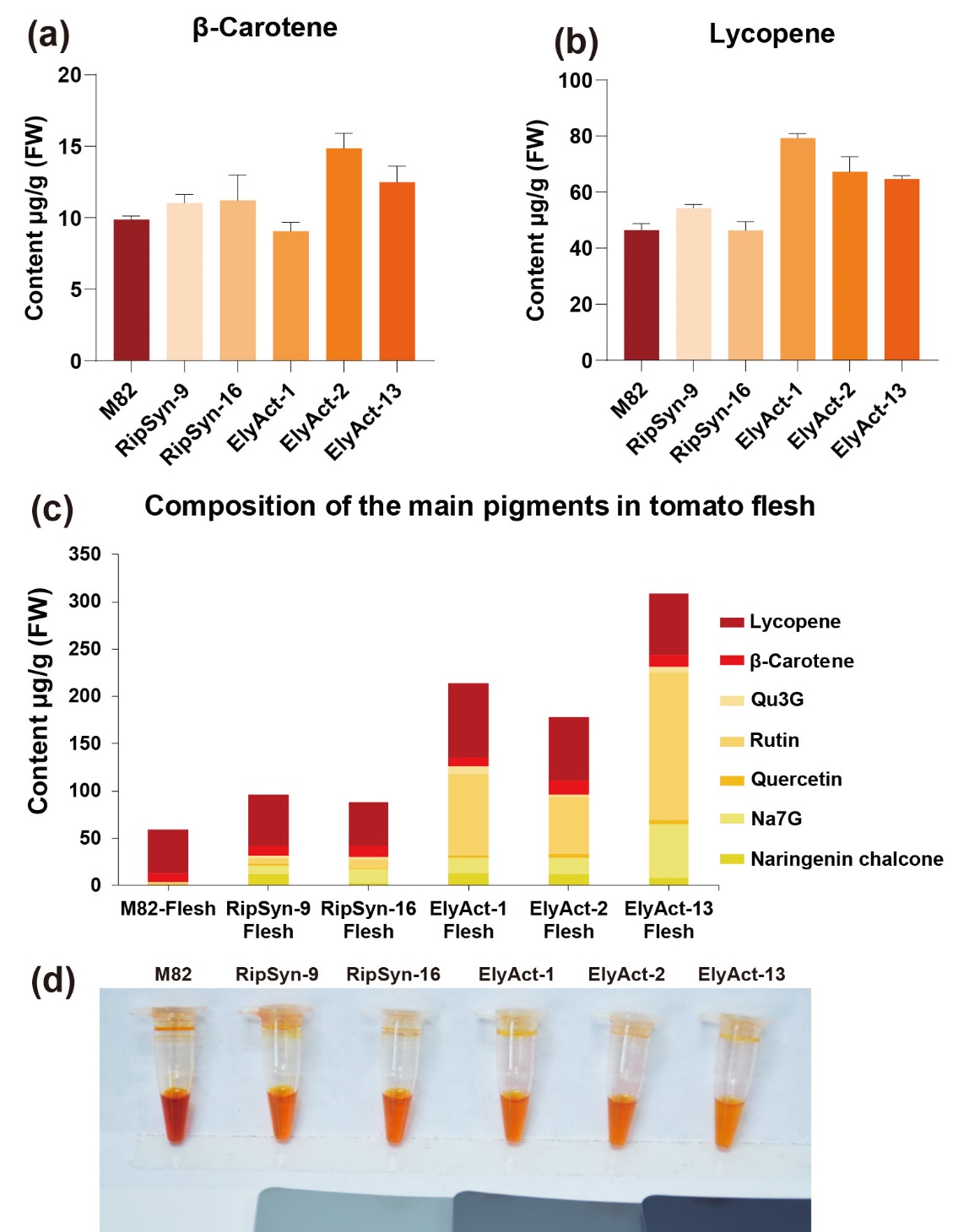


**Supplementary Figure S13. *In vitro* recapitulation of the fruit color of transgenic tomatoes with elevated flavonol levels.**

(a) Lycopene content in the tomato flesh of different transgenic lines. (b) β-carotene content in the tomato flesh of different transgenic lines. (c) (c) Concentration of flavonol and carotenoid pigments (μg/g fresh weight) in tomato fruit flesh of different transgenic lines. Qu3G (quercetin 3-*O*-glucoside), Na7G (naringenin 7-*O*-glucoside). (d) Reproducing the fruit coloration *in vitro* by blending standard solutions of flavonols and carotenoids in ratios reflecting their concentrations in 0.5 g of fresh fruit.


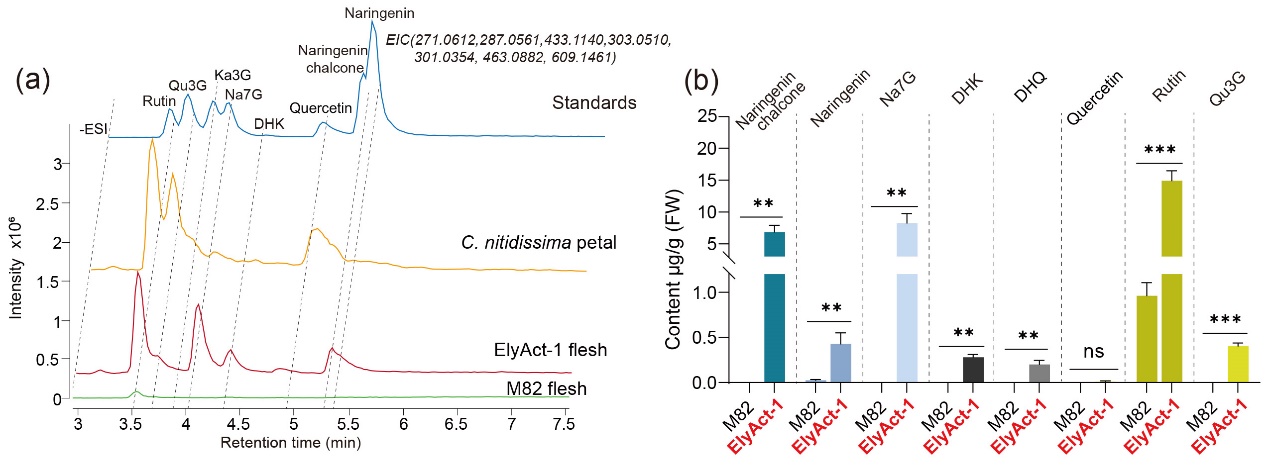


**Supplementary Figure S14. LC/MS profiling of flavonoids in transgenic tomato fruit flesh.**

(a) LC/MS characterization of the fruit flesh from the transgenic tomato lines revealed a complex variety of flavonoid compounds, which eluted concurrently with flavonoids extracted from *C. nitidissima* petals. Displayed are the representative extracted ion chromatograms for specific compounds: Na7G (naringenin 7-*O*-glucoside), DHK (dihydrokaempferol), DHQ (dihydroquercetin), DHQ3G (dihydroquercetin 3-glucoside), Ka3G (kaempferol-3-glucoside), and Qu3G (quercetin 3-*O*-glucoside). (b) Quantification of each flavonoid is provided. Data represent mean ± SEM from three biological replicates. Statistical significance between control and experimental groups was assessed using an unpaired t-test; significance levels are indicated by asterisks (*p<0.05, **p<0.01, ***p<0.001).


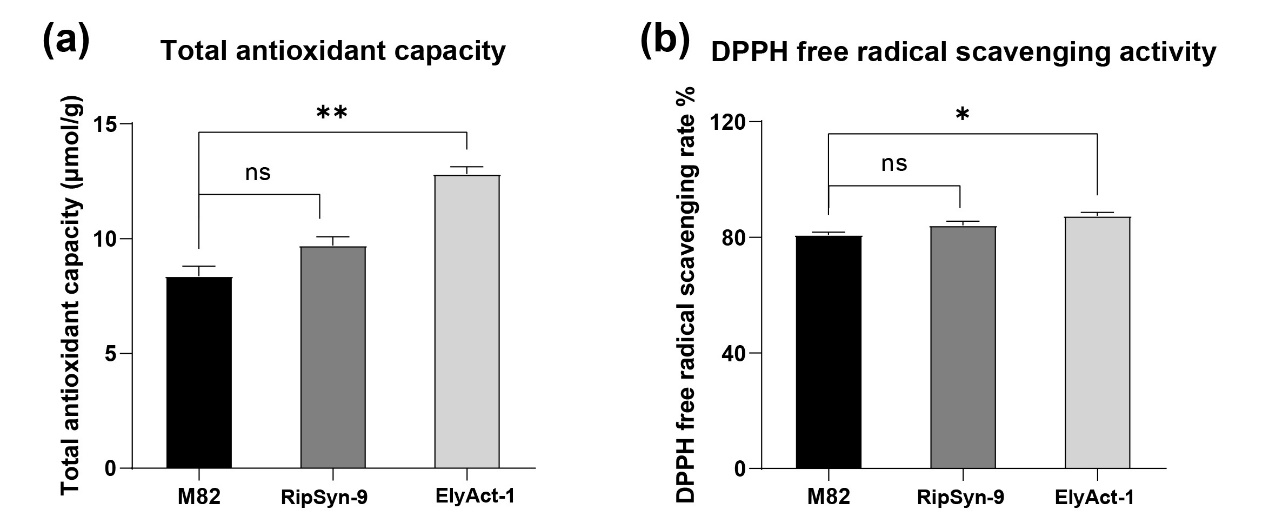


**Supplementary Figure S15. Antioxidant capacity assessment in transgenic tomato fruit flesh.**

(a) Evaluation of the total oxidation capacity (TOC) among fruit flesh of ElyAct-1 line (with high flavonoid content), RipSyn-9 line (with medium flavonoid content), and the control M82 tomatoes. (b) Compares the DPPH free radical scavenging ability in the fruit flesh of ElyAct-1 line, RipSyn-9 line, and control M82. Data are expressed as mean ± SEM from trio of biological replicates. An unpaired t-test was implemented to charaterize statistical significance between control and experimental groups, with significance levels marked by asterisks (*p<0.05, **p<0.01, ***p<0.001).
