# Supplementary material for: Elucidation of the key flavonol biosynthetic pathway in golden Camellia and its application in genetic modification of tomato fruit metabolism
Source: Hortic Res. 2024 Nov 7;12(2):uhae308. doi: 10.1093/hr/uhae308 (PMC11818005; doi:10.1093/hr/uhae308)
Supplement: Web_Material_uhae308 [file web_material_uhae308.zip › Supplementary Data S4.docx]

a #The codes for Matlab software#

[m,n] = size(GENE);

Cor = zeros(m, 1);

Pva = zeros(m, 1);

for i = 1:m

c = GENE(i, :);

[Rco, Pval] = corrcoef(Qu7G, c);

Cor(i, 1) = Rco(1, 2);

Pva(i, 1) = Pval(1, 2);

end

b #The codes for WGCNA analysis#

# Load the WGCNA package

library(WGCNA)

################################input data##########################

# The following setting is important, do not omit.

options(stringsAsFactors = FALSE)

#Read in the female liver data set

fpkm = read.table("Data S1.txt",header=T,comment.char = "",check.names=F)

# Take a quick look at what is in the data set

dim(fpkm)

names(fpkm)

datExpr0 = as.data.frame(t(fpkm[,-1]))

names(datExpr0) = fpkm$ID

rownames(datExpr0) = names(fpkm[,-1])

##check missing value

gsg = goodSamplesGenes(datExpr0, verbose = 3)

gsg$allOK

if (!gsg$allOK)

{

# Optionally, print the gene and sample names that were removed:

if (sum(!gsg$goodGenes)>0)

printFlush(paste("Removing genes:", paste(names(datExpr0)[!gsg$goodGenes], collapse = ", ")))

if (sum(!gsg$goodSamples)>0)

printFlush(paste("Removing samples:", paste(rownames(datExpr0)[!gsg$goodSamples], collapse = ", ")))

# Remove the offending genes and samples from the data:

datExpr0 = datExpr0[gsg$goodSamples, gsg$goodGenes]

}

##filter

meanFPKM=0.5

n=nrow(datExpr0)

datExpr0[n+1,]=apply(datExpr0[c(1:nrow(datExpr0)),],2,mean)

datExpr0=datExpr0[1:n,datExpr0[n+1,] > meanFPKM] # for meanFpkm in row n+1 and it must be above what you set--select meanFpkm>opt$meanFpkm(by rp)

filtered_fpkm=t(datExpr0)

filtered_fpkm=data.frame(rownames(filtered_fpkm),filtered_fpkm)

names(filtered_fpkm)[1]="sample"

head(filtered_fpkm)

write.table(filtered_fpkm, file="FPKM_filter.xls",row.names=F, col.names=T,quote=FALSE,sep="\t")

###############################Sample cluster##############################

sampleTree = hclust(dist(datExpr0), method = "average")

# Plot the sample tree: Open a graphic output window of size 12 by 9 inches

# The user should change the dimensions if the window is too large or too small.

#sizeGrWindow(12,9)

pdf(file = "1_sampleClustering.pdf", width = 12, height = 9)

par(cex = 0.6)

par(mar = c(0,4,2,0))

plot(sampleTree, main = "Sample clustering to detect outliers", sub="", xlab="", cex.lab = 1.5,

cex.axis = 1.5, cex.main = 2)

### Plot a line to show the cut

##abline(h = 26000, col = "red")

dev.off()

##############

###Determine cluster under the line

##clust = cutreeStatic(sampleTree, cutHeight = 26000, minSize = 10)

##table(clust)

###clust 1 contains the samples we want to keep.

##keepSamples = (clust==1)

##datExpr0 = datExpr0[keepSamples, ]

############### ## input trait data###############

#Loading clinical trait data

traitData = read.table("Data S2.txt",row.names=1,header=T,comment.char = "",check.names=F)

dim(traitData)

names(traitData)

# remove columns that hold information we do not need.

allTraits = traitData

dim(allTraits)

names(allTraits)

# Form a data frame analogous to expression data that will hold the clinical traits.

fpkmSamples = rownames(datExpr0)

traitSamples =rownames(allTraits)

traitRows = match(fpkmSamples, traitSamples)

datTraits = allTraits[traitRows,]

rownames(datTraits)

collectGarbage()

# Re-cluster samples

sampleTree2 = hclust(dist(datExpr0), method = "average")

# Convert traits to a color representation: white means low, red means high, grey means missing entry

traitColors = numbers2colors(datTraits, signed = FALSE)

# Plot the sample dendrogram and the colors underneath.

#sizeGrWindow(12,12)

pdf(file="2_Sample dendrogram and trait heatmap.pdf",width=12,height=12)

plotDendroAndColors(sampleTree2, traitColors,

groupLabels = names(datTraits),

main = "Sample dendrogram and trait heatmap")

dev.off()

##################################

save(datExpr0, file = "fpkm_forAnalysis.RData")

save(datTraits, file="trait_forAnalysis.RData")

#############################network constr########################################

# Allow multi-threading within WGCNA. At present this call is necessary.

# Any error here may be ignored but you may want to update WGCNA if you see one.

# Caution: skip this line if you run RStudio or other third-party R environments.

# See note above.

enableWGCNAThreads()

# Choose a set of soft-thresholding powers

powers = c(1:30)

# Call the network topology analysis function

sft = pickSoftThreshold(datExpr0, powerVector = powers, verbose = 5)

# Plot the results:

#sizeGrWindow(9, 5)

pdf(file="3_Scale independence.pdf",width=9,height=5)

par(mfrow = c(1,2))

cex1 = 0.76

# Scale-free topology fit index as a function of the soft-thresholding power

plot(sft$fitIndices[,1], -sign(sft$fitIndices[,3])*sft$fitIndices[,2],

xlab="Soft Threshold (power)",ylab="Scale Free Topology Model Fit,signed R^2",type="n",

main = paste("Scale independence"));

text(sft$fitIndices[,1], -sign(sft$fitIndices[,3])*sft$fitIndices[,2],

labels=powers,cex=cex1,col="red");

# this line corresponds to using an R^2 cut-off of h

abline(h=0.76,col="red")

# Mean connectivity as a function of the soft-thresholding power

plot(sft$fitIndices[,1], sft$fitIndices[,5],

xlab="Soft Threshold (power)",ylab="Mean Connectivity", type="n",

main = paste("Mean connectivity"))

text(sft$fitIndices[,1], sft$fitIndices[,5], labels=powers, cex=cex1,col="red")

dev.off()

######chose the softPower

softPower =sft$powerEstimate

adjacency = adjacency(datExpr0, power = softPower)

##### Turn adjacency into topological overlap

TOM = TOMsimilarity(adjacency);

dissTOM = 1-TOM

# Call the hierarchical clustering function

geneTree = hclust(as.dist(dissTOM), method = "average");

# Plot the resulting clustering tree (dendrogram)

#sizeGrWindow(12,9)

pdf(file="4_Gene clustering on TOM-based dissimilarity.pdf",width=12,height=9)

plot(geneTree, xlab="", sub="", main = "Gene clustering on TOM-based dissimilarity",

labels = FALSE, hang = 0.04)

dev.off()

# We like large modules, so we set the minimum module size relatively high:

minModuleSize = 100

# Module identification using dynamic tree cut:

dynamicMods = cutreeDynamic(dendro = geneTree, distM = dissTOM,

deepSplit = 2, pamRespectsDendro = FALSE,

minClusterSize = minModuleSize);

table(dynamicMods)

# Convert numeric lables into colors

dynamicColors = labels2colors(dynamicMods)

table(dynamicColors)

# Plot the dendrogram and colors underneath

#sizeGrWindow(8,6)

pdf(file="5_Dynamic Tree Cut.pdf",width=8,height=6)

plotDendroAndColors(geneTree, dynamicColors, "Dynamic Tree Cut",

dendroLabels = FALSE, hang = 0.03,

addGuide = TRUE, guideHang = 0.05,

main = "Gene dendrogram and module colors")

dev.off()

# Calculate eigengenes

MEList = moduleEigengenes(datExpr0, colors = dynamicColors)

MEs = MEList$eigengenes

# Calculate dissimilarity of module eigengenes

MEDiss = 1-cor(MEs);

# Cluster module eigengenes

METree = hclust(as.dist(MEDiss), method = "average")

# Plot the result

#sizeGrWindow(7, 6)

pdf(file="6_Clustering of module eigengenes.pdf",width=7,height=6)

plot(METree, main = "Clustering of module eigengenes",

xlab = "", sub = "")

MEDissThres = 0.25

# Plot the cut line into the dendrogram

abline(h=MEDissThres, col = "red")

dev.off()

# Call an automatic merging function

merge = mergeCloseModules(datExpr0, dynamicColors, cutHeight = MEDissThres, verbose = 3)

# The merged module colors

mergedColors = merge$colors

# Eigengenes of the new merged modules:

mergedMEs = merge$newMEs

#sizeGrWindow(12, 9)

pdf(file="7_merged dynamic.pdf", width = 9, height = 6)

plotDendroAndColors(geneTree, cbind(dynamicColors, mergedColors),

c("Dynamic Tree Cut", "Merged dynamic"),

dendroLabels = FALSE, hang = 0.03,

addGuide = TRUE, guideHang = 0.05)

dev.off()

# Rename to moduleColors

moduleColors = mergedColors

# Construct numerical labels corresponding to the colors

colorOrder = c("grey", standardColors(50))

moduleLabels = match(moduleColors, colorOrder)-1

MEs = mergedMEs

# Save module colors and labels for use in subsequent parts

save(MEs, TOM, dissTOM, moduleLabels, moduleColors, geneTree, sft, file = "networkConstruction-stepByStep.RData")

###########################relate modules to external clinical triats##############################

# Define numbers of genes and samples

nGenes = ncol(datExpr0)

nSamples = nrow(datExpr0)

moduleTraitCor = cor(MEs, datTraits, use = "p")

moduleTraitPvalue = corPvalueStudent(moduleTraitCor, nSamples)

#sizeGrWindow(10,6)

pdf(file="8_Module-trait relationships.pdf",width=10,height=6)

# Will display correlations and their p-values

textMatrix = paste(signif(moduleTraitCor, 2), "\n(",

signif(moduleTraitPvalue, 1), ")", sep = "")

dim(textMatrix) = dim(moduleTraitCor)

par(mar = c(6, 8.5, 3, 3))

# Display the correlation values within a heatmap plot

labeledHeatmap(Matrix = moduleTraitCor,

xLabels = names(datTraits),

yLabels = names(MEs),

ySymbols = names(MEs),

colorLabels = FALSE,

colors = greenWhiteRed(50),

textMatrix = textMatrix,

setStdMargins = FALSE,

cex.text = 0.5,

zlim = c(-1,1),

main = paste("Module-trait relationships"))

dev.off()

######## Define variable weight containing all column of datTraits

###MM and GS

# names (colors) of the modules

modNames = substring(names(MEs), 3)

geneModuleMembership = as.data.frame(cor(datExpr0, MEs, use = "p"))

MMPvalue = as.data.frame(corPvalueStudent(as.matrix(geneModuleMembership), nSamples))

names(geneModuleMembership) = paste("MM", modNames, sep="")

names(MMPvalue) = paste("p.MM", modNames, sep="")

#names of those trait

traitNames=names(datTraits)

geneTraitSignificance = as.data.frame(cor(datExpr0, datTraits, use = "p"))

GSPvalue = as.data.frame(corPvalueStudent(as.matrix(geneTraitSignificance), nSamples))

names(geneTraitSignificance) = paste("GS.", traitNames, sep="")

names(GSPvalue) = paste("p.GS.", traitNames, sep="")

####plot MM vs GS for each trait vs each module

##########example:royalblue and CK

#module="royalblue"

#column = match(module, modNames)

#moduleGenes = moduleColors==module

#trait="CK"

#traitColumn=match(trait,traitNames)

#sizeGrWindow(7, 7)

#par(mfrow = c(1,1))

#verboseScatterplot(abs(geneModuleMembership[moduleGenes, column]),

abs(geneTraitSignificance[moduleGenes, traitColumn]),

xlab = paste("Module Membership in", module, "module"),

ylab = paste("Gene significance for ",trait),

main = paste("Module membership vs. gene significance\n"),

cex.main = 1.2, cex.lab = 1.2, cex.axis = 1.2, col = module)

######

for (trait in traitNames){

traitColumn=match(trait,traitNames)

for (module in modNames){

column = match(module, modNames)

moduleGenes = moduleColors==module

if (nrow(geneModuleMembership[moduleGenes,]) > 1){

#sizeGrWindow(7, 7)

pdf(file=paste("9_", trait, "_", module,"_Module membership vs gene significance.pdf",sep=""),width=7,height=7)

par(mfrow = c(1,1))

verboseScatterplot(abs(geneModuleMembership[moduleGenes, column]),

abs(geneTraitSignificance[moduleGenes, traitColumn]),

xlab = paste("Module Membership in", module, "module"),

ylab = paste("Gene significance for ",trait),

main = paste("Module membership vs. gene significance\n"),

cex.main = 1.2, cex.lab = 1.2, cex.axis = 1.2, col = module)

dev.off()

}

}

}

#####

names(datExpr0)

probes = names(datExpr0)

#################export GS and MM###############

geneInfo0 = data.frame(probes= probes,

moduleColor = moduleColors)

for (Tra in 1:ncol(geneTraitSignificance))

{

oldNames = names(geneInfo0)

geneInfo0 = data.frame(geneInfo0, geneTraitSignificance[,Tra],

GSPvalue[, Tra])

names(geneInfo0) = c(oldNames,names(geneTraitSignificance)[Tra],

names(GSPvalue)[Tra])

}

for (mod in 1:ncol(geneModuleMembership))

{

oldNames = names(geneInfo0)

geneInfo0 = data.frame(geneInfo0, geneModuleMembership[,mod],

MMPvalue[, mod])

names(geneInfo0) = c(oldNames,names(geneModuleMembership)[mod],

names(MMPvalue)[mod])

}

geneOrder =order(geneInfo0$moduleColor)

geneInfo = geneInfo0[geneOrder, ]

write.table(geneInfo, file = "10_GS_and_MM.xls",sep="\t",row.names=F)

####################################Visualizing the gene network#############################

nGenes = ncol(datExpr0)

nSamples = nrow(datExpr0)

# Transform dissTOM with a power to make moderately strong connections more visible in the heatmap

plotTOM = dissTOM^7

# Set diagonal to NA for a nicer plot

diag(plotTOM) = NA

# Call the plot function

#sizeGrWindow(9,9)

pdf(file="12_Network heatmap plot_all gene.pdf",width=9, height=9)

TOMplot(plotTOM, geneTree, moduleColors, main = "Network heatmap plot, all genes")

dev.off()

nSelect = 400

# For reproducibility, we set the random seed

set.seed(10)

select = sample(nGenes, size = nSelect)

selectTOM = dissTOM[select, select]

# There's no simple way of restricting a clustering tree to a subset of genes, so we must re-cluster.

selectTree = hclust(as.dist(selectTOM), method = "average")

selectColors = moduleColors[select]

# Open a graphical window

#sizeGrWindow(9,9)

# Taking the dissimilarity to a power, say 10, makes the plot more informative by effectively changing

# the color palette; setting the diagonal to NA also improves the clarity of the plot

plotDiss = selectTOM^7

diag(plotDiss) = NA

pdf(file="13_Network heatmap plot_selected genes.pdf",width=9, height=9)

TOMplot(plotDiss, selectTree, selectColors, main = "Network heatmap plot, selected genes")

dev.off()

#################################Visualizing the gene network of eigengenes#######################

#sizeGrWindow(5,7.5)

pdf(file="14_Eigengene dendrogram and Eigengene adjacency heatmap.pdf", width=5, height=7.5)

par(cex = 0.9)

plotEigengeneNetworks(MEs, "", marDendro = c(0,4,1,2), marHeatmap = c(3,4,1,2), cex.lab = 0.8, xLabelsAngle= 90)

dev.off()

#or devide into two parts

# Plot the dendrogram

#sizeGrWindow(6,6);

pdf(file="15_Eigengene dendrogram_2.pdf",width=6, height=6)

par(cex = 1.0)

plotEigengeneNetworks(MEs, "Eigengene dendrogram", marDendro = c(0,4,2,0), plotHeatmaps = FALSE)

dev.off()

pdf(file="15_Eigengene adjacency heatmap_2.pdf",width=6, height=6)

# Plot the heatmap matrix (note: this plot will overwrite the dendrogram plot)

par(cex = 1.0)

plotEigengeneNetworks(MEs, "Eigengene adjacency heatmap", marHeatmap = c(3,4,2,2), plotDendrograms = FALSE, xLabelsAngle = 90)

dev.off()

###########################Exporting to Cytoscape all one by one ##########################

# Select each module

for (mod in 1:nrow(table(moduleColors)))

{

modules = names(table(moduleColors))[mod]

# Select module probes

probes = names(datExpr0)

inModule = (moduleColors == modules)

modProbes = probes[inModule]

modGenes = modProbes

# Select the corresponding Topological Overlap

modTOM = TOM[inModule, inModule]

dimnames(modTOM) = list(modProbes, modProbes)

# Export the network into edge and node list files Cytoscape can read

cyt = exportNetworkToCytoscape(modTOM,

edgeFile = paste("CytoscapeInput-edges-", modules , ".txt", sep=""),

nodeFile = paste("CytoscapeInput-nodes-", modules, ".txt", sep=""),

weighted = TRUE,

threshold = 0.02,

nodeNames = modProbes,

altNodeNames = modGenes,

nodeAttr = moduleColors[inModule])

}
